# Multiplexed Profiling of Individual Extracellular Vesicles Reveals Lung Cancer‐Associated Protein Signatures

**DOI:** 10.1002/advs.77301

**Published:** 2026-08-25

**Authors:** Mi Hyeon Cho, Yein Chung, Yoonjeong Choi, Baekdong Cha, Jayeon Song, Nuri Oh, Ala Jo, Thomas S. C. Ng, Hyunho Kim, Hyun‐Kyung Woo, Chang Hyun Kim, Cesar M. Castro, Miles A. Miller, Hakho Lee

**Affiliations:** ^1^ Center For Systems Biology Massachusetts General Hospital Research Institute Boston Massachusetts USA; ^2^ Department of Radiology, Harvard Medical School Massachusetts General Hospital Boston Massachusetts USA; ^3^ Metropolitan Seoul Center Korea Basic Science Institute (KBSI) Seoul Republic of Korea; ^4^ Department of Immunology University of Toronto Toronto Ontario Canada; ^5^ Department of Industrial Machinery DX. Korea Institute of Machinery & Materials (KIMM) Daejeon Republic of Korea; ^6^ Department of Physiology, College of Medicine Ewha Womans University Seoul Republic of Korea; ^7^ Inflammation‐Cancer Microenvironment Research Center, College of Medicine Ewha Womans University Seoul Republic of Korea; ^8^ Department of Surgery Chonnam National University Hwasun Hospital and Medical School Jeonnam Republic of Korea; ^9^ Department of Medicine, Massachusetts General Hospital Harvard Medical School Boston Massachusetts USA

**Keywords:** biotechnology, extracellular vesicle, lung cancer

## Abstract

Lung cancer is frequently diagnosed after curative treatment windows have narrowed, creating a need for minimally invasive biomarkers that report tumor development at earlier stages. Extracellular vesicles (EVs) are promising analytical targets because they carry molecular cargo from their originating cells, but tumor‐associated EV signals can be rare in blood and obscured in bulk measurements. Here, we present **Cygnus** (**Cy**clic‐imaging **g**ateway to **n**anovesicles **u**nderlying **s**ignature), an end‐to‐end workflow that integrates cyclic immunofluorescence imaging with multiscale analysis of individual EVs. Cygnus preserves vesicle‐level measurements, quantifies marker co‐expression, resolves EV subpopulations, and summarizes single‐vesicle phenotypes into sample‐level profiles. In a genetically engineered mouse model of lung adenocarcinoma, Cygnus revealed dynamic changes in EV protein composition and identified an EpCAM‐ and/or CTSH‐positive EV subpopulation before tumors were detectable by radiography. In a pilot clinical cohort (*n* = 35), plasma EV profiling showed lung cancer‐associated marker patterns compared with non‐cancer controls and nominated a CTSH/PDL1/MET marker combination for future validation. These findings support multiplexed individual‐EV profiling as a strategy for defining candidate lung cancer‐associated EV signatures and position Cygnus as an integrated workflow for translating vesicle‐level heterogeneity into sample‐level EV profiles.

## Graphical abstract

Cygnus (**Cy**clic‐imaging **g**ateway to **n**anovesicles **u**nderlying **s**ignature) integrates multiplexed single‐EV protein imaging with a practical analysis workflow to examine EV phenotypes at vesicle, subpopulation, and sample scales.

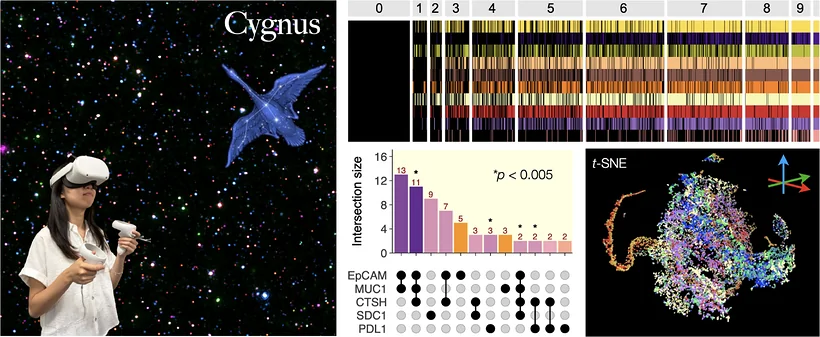

## 1 Introduction

Lung cancer (LC) is frequently asymptomatic during early disease development and is often diagnosed after curative treatment options have narrowed. Liquid biopsies can offer a sensitive, minimally invasive means to detect LC‐associated molecular changes before overt radiographic detection. Extracellular vesicles (EVs), in particular, are impactful analytical targets [1, 2, 3]. These nanoscale particles carry a diverse repertoire of molecular cargo, including proteins, mRNA, and microRNA, and reflect the physiological and pathological states of their parent cells [4, 5]. Tumor‐associated EVs have been shown to serve as surrogate biomarkers for cancer detection and monitoring [6, 7, 8, 9, 10, 11, 12, 13]. Among EV‐derived analytes, proteins are attractive targets, as their expression patterns can enable tumor detection with high specificity, inform tissue or subtype origin, and distinguish indolent from aggressive disease states [14, 15, 16].

Despite these encouraging results, technical challenges remain when using EV analyses for early LC diagnostics. Tumor‐derived EVs constitute only a small fraction (<5%) of total EVs in blood [17], whereas normal host cell‐derived vesicles account for the vast majority of circulating vesicles. This imbalance is pronounced during early cancer development, when tumor‐associated EVs are scarce due to low tumor burden. Conventional EV assays, which require large numbers of EVs (>10,000), are thus limited in distinguishing true tumor signal from background vesicle populations and defining EV profiles that arise during early cancer development [18, 19, 20, 21].

To overcome these challenges, EV protein analysis has increasingly shifted toward single‐vesicle analysis [22, 23, 24, 25], which can detect rare EV populations and interrogate molecular co‐expression at the individual vesicle level. Recent advances in imaging, including cyclic immunofluorescence microscopy [19, 20] and super‐resolution microscopy [26, 27], have enabled the generation of large‐scale single‐EV datasets, paving the way for systematic classification of vesicle subpopulations and the discovery of early disease‐associated phenotypes.

Here, we present Cygnus (**Cy**clic‐imaging **g**ateway to **n**anovesicles **u**nderlying **s**ignature), an end‐to‐end workflow that integrates cyclic immunofluorescence microscopy with multiscale single‐EV analysis for early LC detection (Scheme 1). The workflow enables i) multi‐marker (>12 proteins) profiling on individual EVs; ii) image alignment and EV detection; iii) multiscale analyses spanning marker co‐expression, EV subpopulation mapping, and sample‐level summaries; and iv) distillation of single‐EV measurements into quantitative features compatible with bulk EV assays.

**SCHEME 1:**
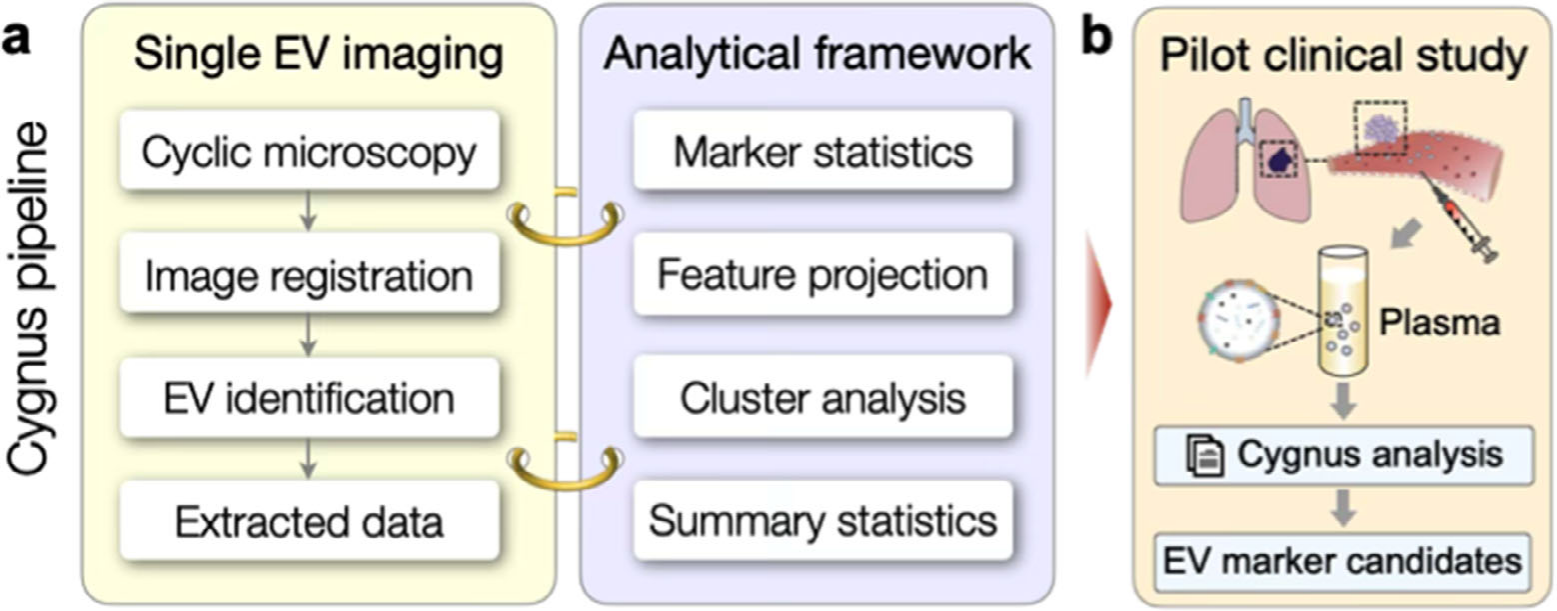
Cyclic‐imaging gateway to nanovesicles underlying signature (Cygnus). (a) Cygnus integrates multiplexed single‐EV imaging with multiscale data analysis. Cyclic immunofluorescence microscopy generates single‐EV protein profiles, which are processed for image alignment, vesicle detection, and intensity extraction. These datasets are analyzed to generate single‐vesicle profiles, marker co‐expression patterns, dimensionally reduced embeddings, and population‐level summaries. (b) In a pilot clinical application, Cygnus was used to profile plasma EVs from non‐cancer controls and patients with early‐ or late‐stage lung cancer, identifying candidate EV signatures for future validation.

We applied this workflow to longitudinal plasma samples from a genetically engineered mouse model of lung adenocarcinoma, which enabled temporal tracking of EV phenotypes during early LC development. We further analyzed single EVs in human plasma samples (*n* = 35) from lung cancer patients and non‐cancer controls, demonstrating translational applicability to clinical specimens. Together, these studies show that multiscale single‐EV analysis can identify marker co‐expression patterns, resolve disease‐associated EV subpopulations, and translate vesicle‐level phenotypes into sample‐level signatures relevant to early cancer detection and precision oncology.

## 2 Results

### 2.1 Multiplexed Protein Imaging of Individual EVs

We first established a cyclic imaging workflow to profile LC‐associated proteins on individual EVs (Figure 1a; see Methods). EVs were prelabeled with an amine‐reactive fluorescent dye for total protein labeling, which provided localization signals for vesicle identification during image analysis. Labeled EVs were then immobilized on poly‐L‐lysine‐coated glass substrates to maintain their positions across repeated imaging cycles. Each imaging cycle consisted of immunostaining with fluorescently labeled antibodies (Alexa Fluor 488, 555, and 647), image acquisition, and chemical quenching of fluorescence signals using an H_2_O_2_ buffer (3% v/v) [20, 28].

**FIGURE 1:**
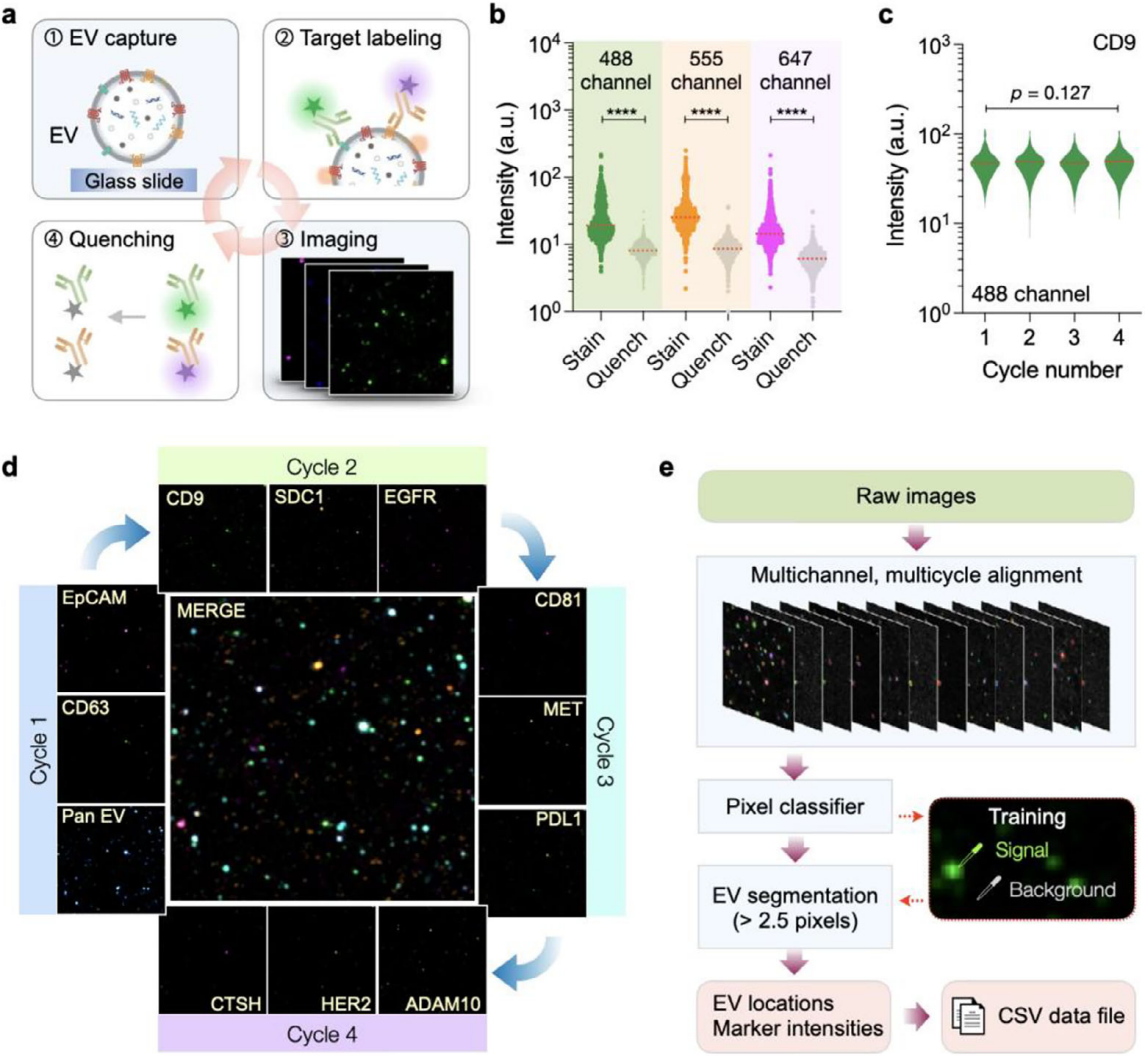
Multiplexed single‐EV imaging in Cygnus. (a) Assay workflow. EVs were labeled with a total protein stain, immobilized on glass, immunostained with fluorescent antibodies, imaged, and quenched (3% v/v H_2_O_2_) before subsequent staining cycles. (b) Fluorescence quenching efficiency across 488, 555, and 647 nm channels. Quenching removed >99% of fluorescence signal (****, *p* < 0.0001). (c) Assessment of cycle‐dependent bias. EVs were immunostained for CD9 in different imaging cycles. Mean fluorescence intensity (MFI) values showed no significant dependence on staining order (*p* = 0.127; one‐way ANOVA). (d) Representative 12‐marker profiling of KP1.9‐derived EVs across four imaging cycles. (e) Image post‐processing and data extraction. Cyclic image stacks were aligned using a custom ImageJ plugin. Individual EVs were then segmented, and marker intensity values were tabulated for downstream analysis.

We assessed EV retention during cyclic processing. Pan‐EV‐positive particle counts remained largely stable across four imaging cycles, indicating no progressive EV loss during repeated staining, imaging, quenching, and washing (Figure S1). For downstream analysis, EV locations were defined from the pan‐EV signal in the first imaging cycle and used as fixed coordinates for marker extraction from registered images in subsequent cycles. The quenching step removed >99% of fluorescence across channels (Figure 1b), enabling subsequent rounds of staining with distinct antibody panels on the same EVs. Single‐fluorophore bleed‐through experiments showed minimal channel crosstalk, with off‐channel signal below the detection threshold under the tested imaging conditions (Figure S2).

We next evaluated potential sources of background signal. Dye‐only, antibody‐only, non‐EV fraction, and blank‐isolation controls produced few detected spots, indicating minimal background from staining or EV‐isolation procedures (Figure S3a). In labeled EV samples, detergent treatment with 1% Triton X‐100 eliminated fluorescent particle signals, supporting that the detected signals originated from membrane‐associated, EV‐like particles rather than dye or antibody aggregates (Figure S3b).

Dilution and spatial‐separation analyses further supported discrete particle detection. In EV‐dilution experiments, detected spot counts decreased with lower EV input, whereas mean per‐spot fluorescence intensity remained largely unchanged (Figure S4). Because EV aggregation is expected to increase per‐spot intensity, particularly at higher EV concentrations, the stable intensity across dilutions supports that most detected spots were discrete, EV‐associated particles. Nearest‐neighbor analysis further showed that >99% of detected spots were separated by more than the diffraction limit (≈0.6 µm; Figure S5), indicating that most spots were spatially resolved under the imaging conditions used.

We also assessed whether repeated staining and quenching introduced cycle‐dependent bias. EV aliquots were stained for CD9 at different imaging cycles, and mean fluorescence intensity (MFI) values showed no significant dependence on cycle number (*p* = 0.127; one‐way ANOVA; Figure 1c). We then extended this analysis to other LC‐associated markers, CTSH, PDL1, MET, and EpCAM, whose MFI values also showed no significant cycle‐dependent changes (*p* > 0.05, one‐way ANOVA; Figure S6).

To estimate false co‐localization caused by random spatial overlap, EV preparations were separately labeled with red or green pan‐EV dyes and mixed at defined ratios. False co‐localization, measured as red/green double‐positive spots, remained low across the tested mixing ratios (≈0.1%–1.3% of detected events; Figure S7), indicating that random overlap contributed minimally under the imaging conditions used in this study.

Figure 1d shows representative cyclic images of EVs derived from KP1.9 mouse lung adenocarcinoma cells. In this experiment, a panel of 12 markers was profiled across four imaging cycles, including canonical EV markers (CD63, CD9, CD81) and eight LC‐associated proteins. EVs from human lung adenocarcinoma cell lines (H2228 and A549) were analyzed using the same approach (Figures S8 and S9), demonstrating the applicability of the imaging strategy across model systems.

### 2.2 Image Processing Pipeline

Acquired images were subsequently processed to extract vesicle‐level marker intensities (Figure 1e). To facilitate this process, we developed custom ImageJ plugins for image alignment (Note S1). These plugins corrected translational and rotational offsets across cycles, ensuring accurate spatial registration of fluorescence signals from the same EV (Movie S1). Individual EVs were then segmented using a trained pixel classifier, which yielded consistent results across technical replicates (Figure S10). These steps transformed raw microscopy images into structured vesicle‐level datasets containing marker measurements and metadata for downstream multiscale analysis.

### 2.3 Computational Pipeline for Multiscale Analysis of Single‐EV Protein Profiles

We next designed a computational pipeline to process large‐scale, high‐dimensional single‐EV datasets. The key goal was to enable multiscale EV analysis by preserving vesicle identity, distinguishing true marker co‐expression from stochastic overlap, and summarizing vesicle‐level patterns in forms that can be compared across samples. Implemented as an open‐source R package, the pipeline imports vesicle‐level datasets from multiplexed imaging, stores marker measurements and metadata in a unified R object, and applies preprocessing, marker‐centered analysis, EV‐centered analysis, and sample‐level summarization (Figure 2a).

**FIGURE 2:**
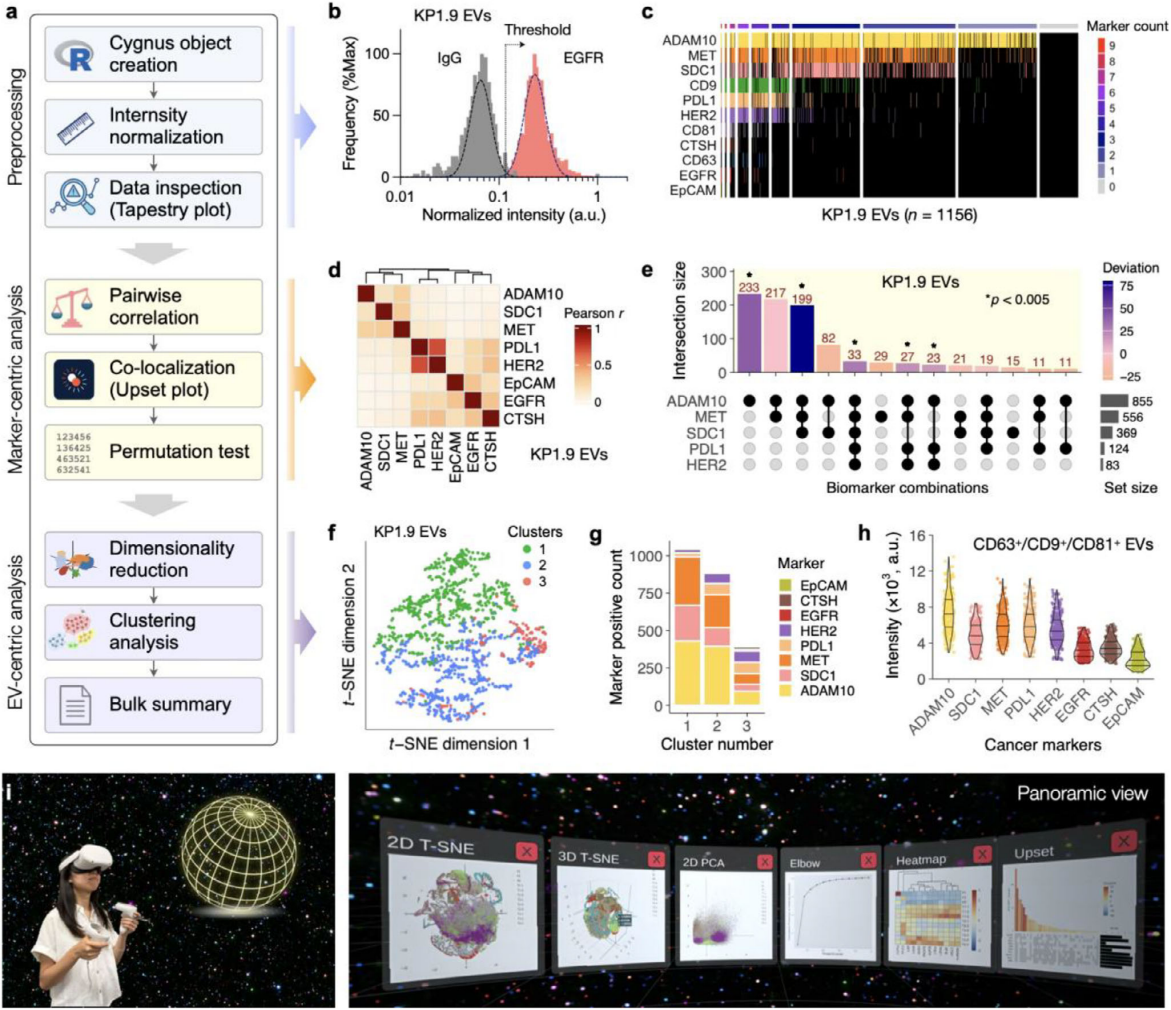
Cygnus framework for multiscale single‐EV analysis. (a) Overall workflow for preprocessing, marker‐centered analysis, EV‐centered analysis, and sample‐level summarization. (b) Marker‐positivity thresholding using isotype IgG controls. Representative EGFR and mouse IgG intensity distributions are shown for KP1.9‐derived EVs. (c) Tapestry plot showing marker‐positivity patterns across individual EVs. Each column represents a single EV, and each row corresponds to an individual marker. (d) Marker‐centered correlation analysis. Pairwise correlations assessed coordinated expression patterns among EV‐associated proteins. (e) UpSet‐based co‐localization analysis. Marker combinations were enumerated, and the number of EVs exhibiting each combination was quantified. Permutation testing identified marker combinations exceeding random overlap (Bonferroni‐adjusted *p* < 0.005). (f) EV‐centered dimensionality reduction. Dimensionality reduction applied to 12‐marker intensity profiles resolved three distinct EV clusters in KP1.9‐derived samples. (g) Major EV clusters from (f) were analyzed for marker composition. (h) Cross‐scale summarization. Marker intensities were obtained for tetraspanin‐positive EVs. (i) Optional CygnusVR display for interactive visualization of raw images and analytical outputs.

The preprocessing module compensates for background and nonspecific signals on a per‐marker basis. Marker‐positivity thresholds are defined using control IgG measurements (Figure 2b), providing an assay‐specific approach to signal discrimination. Additional preprocessing options include intensity normalization for inter‐marker comparisons, scaling relative to a pan‐EV marker to account for vesicle size variability, and converting continuous intensities to binary presence/absence calls. Following preprocessing, Cygnus displays marker‐presence patterns across all EVs using a tapestry‐like plot (Figure 2c). This visualization provides a global overview of EV marker distributions and enables assessment of data quality, batch effects, outliers, and anomalous features before downstream analysis.

Marker‐centered analysis includes per‐marker expression frequency, pairwise correlation (Figure 2d), and higher‐order marker co‐localization. Cygnus uses UpSet analysis to enumerate marker combinations and quantify the number of EVs exhibiting each combination (Figure 2e) [29, 30]. Compared with traditional Venn diagrams (Figure S11), UpSet analysis provides a clearer visualization of co‐expression patterns as the number of markers increases.

We further implemented a permutation‐based statistical test to determine whether observed co‐localization patterns exceeded expectations from random marker coincidence (see Methods). Marker labels were randomly assigned across EVs while preserving each marker's observed expression frequency, generating a null distribution for each marker subset. Observed deviations from the null model were then assigned *p*‐values, with Bonferroni correction applied across evaluated marker combinations [31]. Applied to KP1.9‐derived EVs, this analysis identified statistically significant LC‐associated marker patterns, including EVs expressing ADAM10 alone and EVs co‐expressing ADAM10, MET, and SDC1 (Figure 2e).

EV‐centered analysis determines how marker profiles organize individual vesicles into subpopulations. Marker intensity profiles are used as features for dimensionality reduction and clustering, including PCA, *t*‐SNE, UMAP, KNN, Leiden, and HDBSCAN approaches. In KP1.9‐derived EVs, *t*‐SNE visualization revealed three distinct EV clusters (Figure 2f). Cluster‐level marker analysis showed that two major clusters were enriched for EVs co‐expressing ADAM10, MET, and SDC1 (Figure 2g), consistent with the marker‐centered co‐localization results.

Finally, Cygnus transforms vesicle‐level intensity data into population‐level summaries (Figure 2h). This cross‐scale analysis simulates ensemble EV immunoassays in which vesicles are captured based on selected markers and probed for target protein expression. These summaries connect single‐EV phenotypes to bulk EV assay readouts, supporting translation of vesicle‐level discoveries into practical diagnostic features.

Cygnus further includes an optional VR display module for exploratory visualization of raw images and analytical outputs (Figure 2i and Figure S12). This module was designed as an interpretive aid, allowing users to explore complex data structures using spatial cues that complement conventional two‐dimensional displays. The application was implemented as standalone software (see Methods) and renders Cygnus analytical outputs in real time (Movie S2).

### 2.4 Cygnus Single‐EV Profiling in a Mouse LC Model

Having established its imaging and analysis workflow, we next applied Cygnus to early LC detection. We first selected candidate protein markers using RNA sequencing data of tumor and normal lung tissues from the *Kras* ^G12D/+^; *Trp53* ^−/−^ (KP) model [32], the same lung cancer model used for longitudinal plasma EV profiling in this study. Genes overexpressed in early LC compared with normal lung were filtered for membrane localization, transmembrane domains, prior EV annotation, LC relevance, and antibody availability (Figure S13; details in Methods). This strategy nominated EpCAM, CTSH, SDC1, MET, and MUC1 as early LC candidates. We further included EGFR, PDL1, HER2, ADAM10, and SP‐B based on LC biomarker databases, in‐house profiling, and literature review [33, 34, 35, 36, 37].

Using this KP model, we initiated lung tumors by intratracheal Ad‐Cre administration, which activates *Kras* ^G12D^ expression and deletes *Trp53* in lung epithelial cells [38] (Figure 3a). Serial blood samples were collected from individual mice (*n* = 9) one week before tumor induction and weekly thereafter. Tumor progression was monitored by histology, Kras^G12D^ immunohistochemistry, and micro‐computed tomography (µCT). Kras^G12D^‐positive epithelial foci were detected at week 1 and increased over time (Figure S14), consistent with progressive expansion of oncogene‐initiated lung epithelial lesions. Histologic lesions, characterized by atypical adenomatous hyperplasia or small adenomas with uniform nuclear morphology, were observed at week 3 (Figure 3b). Under µCT imaging (Figure 3b, bottom), detectable lesions emerged approximately four weeks after tumor induction (Figure 3c and Figure S15).

**FIGURE 3:**
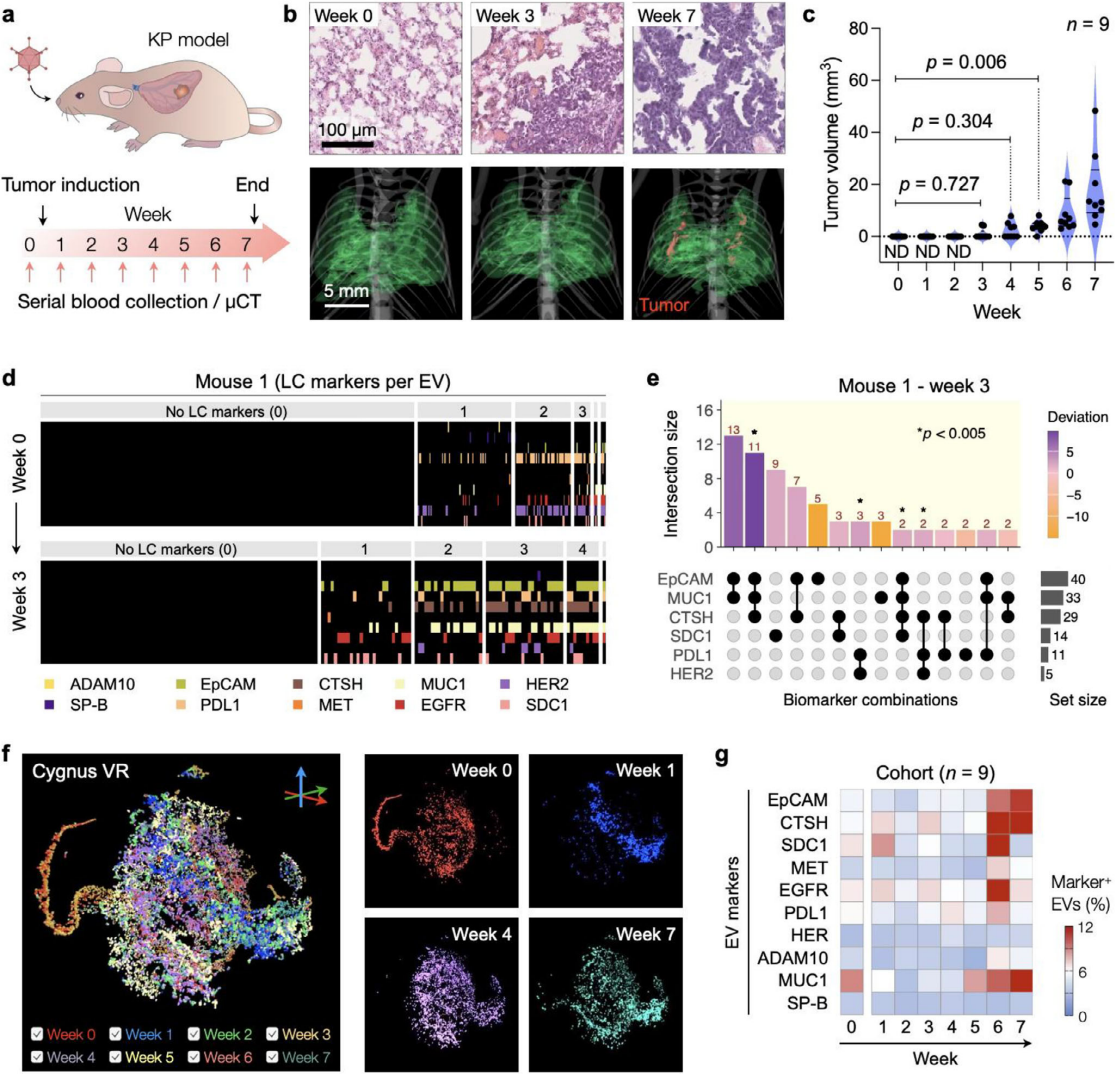
Cygnus single‐EV profiling in a mouse LC model. (a) A Cre‐inducible *Kras* ^G12D/+^; *Trp53* ^–/–^ (KP) mouse model was used to model lung cancer (LC) development. Serial blood samples were collected before tumor induction, and weekly thereafter; micro‐computed tomography (µCT) imaging was performed after each blood draw. (b) Histological analysis of lung tissue (top) confirmed progressive tumor growth over time. µCT imaging (bottom) detected tumor burden at the late stage of tumor development. (c) Tumor volumes were estimated from weekly µCT images of tumor‐bearing mice (*n* = 9). Statistically significant increases in tumor volume were detected approximately five weeks after tumor induction (*p* < 0.05; Sidak multiple‐comparisons test). (d) Tapestry plots revealed a progressive increase in the number of EVs expressing LC‐associated markers during tumor development. (e) In UpSet and permutation analysis, marker combinations involving EpCAM, CTSH, and MUC1 emerged during tumor progression. (f) Three‐dimensional *t*‐SNE visualization of single‐EV profiles from mice (*n* = 3), showing temporal changes in EV subpopulation structure. (g) Marker‐expression summaries. Six markers (EpCAM, CTSH, SDC1, MET, EGFR, PDL1) showed tumor‐associated temporal trends from the pre‐tumor state to early disease.

Cygnus analysis revealed dynamic changes in EV protein profiles during early tumor development. Tapestry plot visualization indicated a progressive increase in EVs positive for LC‐associated markers over time (Figure 3d). UpSet analysis showed minimal marker expression and negligible co‐localization before tumor induction (week 0; Figure S16). In contrast, statistically significant (*p* < 0.005) marker co‐localization patterns emerged shortly after tumor initiation, involving EpCAM, CTSH, and MUC1 (Figure 3e).

EV‐centered analysis further resolved vesicle subpopulations during early disease development. In particular, exploratory CygnusVR visualization helped identify an EV cluster enriched in week‐1 samples (Figure 3f). Marker analysis showed that this cluster contained EVs expressing EpCAM, CTSH, or both markers (Figure S17). Moreover, EpCAM‐ and CTSH‐positive EV fractions generally increased over time across the Ad‐Cre‐induced cohort, whereas EpCAM‐ and CTSH‐positive EV fractions remained low (<4%) in vehicle‐treated KP mice (Figure S18). Together, these analyses suggest that the week‐1‐enriched EV population may reflect EV changes associated with the early phase of tumor initiation in the Ad‐Cre‐induced KP model. Immunohistochemical staining confirmed the expression of EpCAM and CTSH in tumor lesions at week 3 (Figure S19).

Finally, we summarized the longitudinal data by quantifying the fraction of EVs positive for each LC‐associated marker (Figure 3g). This sample‐level analysis was used to refine the marker panel for clinical plasma profiling. Six markers, EpCAM, CTSH, SDC1, MET, EGFR, and PDL1, were chosen based on tumor‐associated temporal trends.

### 2.5 Pilot Single‐EV Plasma Profiling in Clinical LC Plasma Samples

To evaluate the translational feasibility of the Cygnus workflow, we profiled EVs from plasma samples obtained from treatment‐naive LC patients with early‐stage disease (stages I‐II; *n* = 20) or late‐stage disease (stages III‐IV; *n* = 5), as well as from non‐cancer control donors (*n* = 10) (demographic information in Table S1). Plasma EVs were enriched by dual‐mode chromatography (DMC) to reduce lipoprotein contamination (Figure S20) [39]. In prior validation studies, DMC depleted >99.8% of lipoprotein signals while retaining approximately 34% of the input EV‐associated CD63 signal; the relative CD63, CD9, and CD81 profiles were also maintained after separation, supporting minimal selection bias among the tested tetraspanin‐defined EV populations [39, 40, 41]. In the plasma samples analyzed here, DMC‐processed preparations contained EV‐like particles with the expected size, morphology, and marker profiles, while soluble protein and lipoprotein contaminants were reduced (Figure S20).

Prepared EV samples underwent cyclic immunofluorescence imaging (Figure 4a and Figure S21), including staining for total protein (pan‐EV), combined tetraspanin expression (CD9, CD63, CD81), and the six LC‐associated markers selected from the mouse study (EpCAM, CTSH, SDC1, MET, EGFR, and PDL1).

**FIGURE 4:**
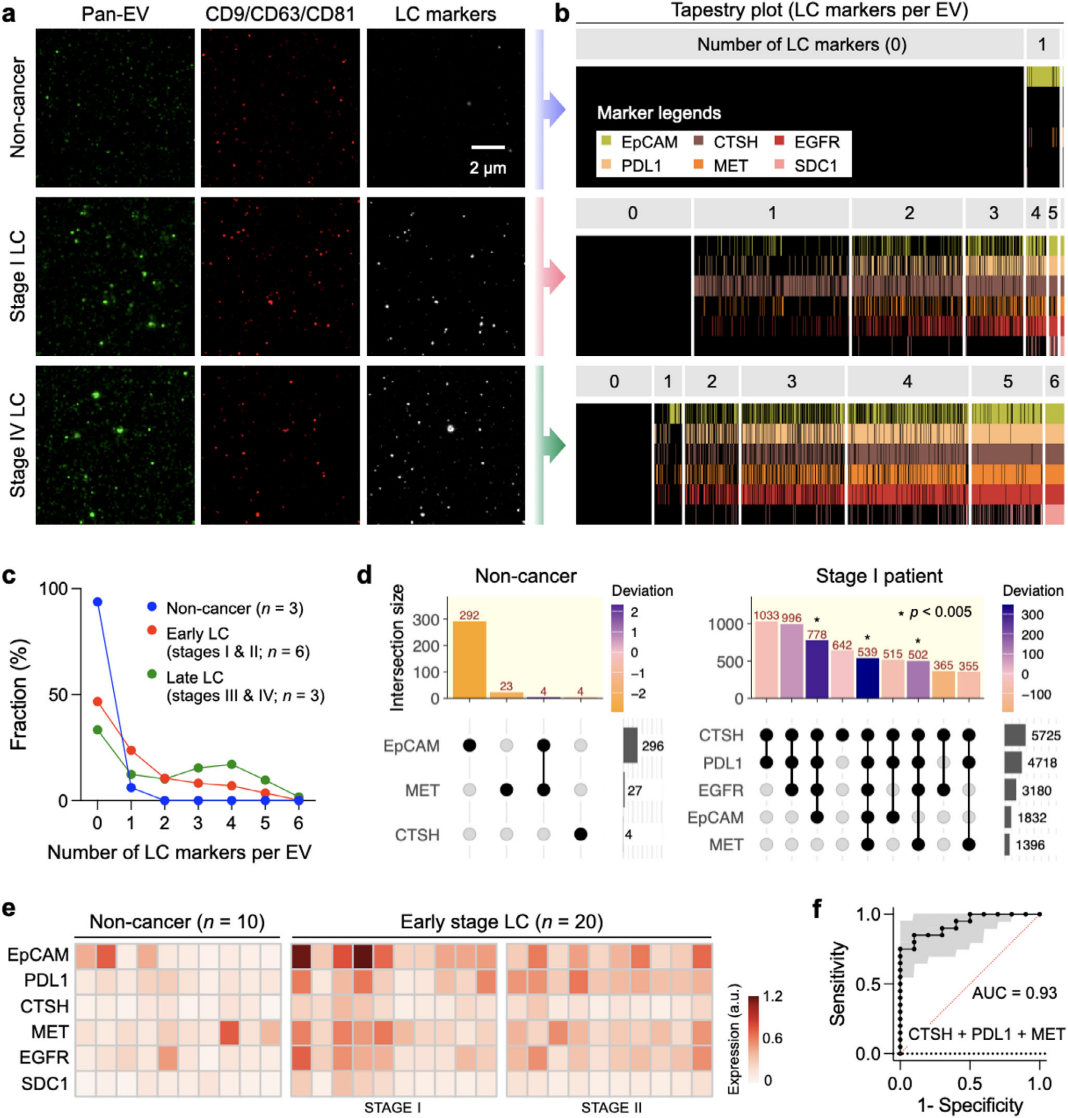
Pilot single‐EV plasma profiling in clinical LC samples. (a) Representative single‐EV fluorescence images from non‐cancer control, stage I LC, and stage IV LC plasma samples. EVs were localized using a pan‐EV stain, defined by tetraspanin staining (CD63/CD81/CD9), and profiled for six LC‐associated markers. (b) Tapestry plots of the representative samples shown in (a). Molecular heterogeneity increased with disease stage. (c) Fractions of EVs expressing different numbers of LC markers. Most EVs (93%) had no LC markers in non‐cancer controls, whereas >50% of EVs from LC patients expressed at least one LC marker. Late‐stage LC samples contained a larger fraction of EVs expressing four or more LC markers compared with early‐stage LC samples. (d) UpSet and permutation analysis showing LC‐associated marker combinations in clinical plasma EVs. Co‐expression involving CTSH and PDL1 emerged as a candidate pattern associated with early LC samples. (e) Marker‐expression summaries of tetraspanin‐positive EVs across clinical cohorts. Marker expression was normalized to tetraspanin levels. Technical replicate (*n* = 3) analysis showed good reproducibility of marker‐positive EV fractions across patient samples and markers (Pearson *r* = 0.88–0.91; Spearman ρ = 0.88–0.92). (f) Exploratory receiver operating characteristic analysis comparing non‐cancer controls (*n* = 10) and early‐stage LC patients (*n* = 20). The CTSH/PDL1/MET marker combination showed the highest classification performance among the tested marker sets within this pilot cohort (AUC, 0.93; 95% confidence interval, 0.85–1.00; DeLong's method). The gray‐shaded region represents the 95% bootstrap confidence interval for sensitivity at fixed specificity values, estimated from 2,000 stratified bootstrap replicates. AUC, area under the curve.

Cygnus‐assisted visualization revealed disease‐associated shifts in single‐EV protein profiles across the clinical cohorts. Tapestry plots showed that most EVs from non‐cancer controls lacked detectable LC marker expression, whereas LC patient samples contained larger fractions of EVs positive for one or more LC‐associated proteins (Figure 4b). Across the cohorts (Figure 4c), the majority (>93%) of EVs were negative for LC marker expression in non‐cancer controls, whereas >50% of EVs from LC patients expressed at least one LC marker. Late‐stage LC samples contained a larger fraction of highly multiplexed vesicles, with 29% of EVs expressing four or more LC markers compared with 10% in early‐stage LC samples.

Consistent with this observation, marker co‐expression analysis suggested increased EV molecular heterogeneity with disease progression. UpSet analysis indicated that LC‐associated marker combinations were uncommon in non‐cancer controls but detectable in patient plasma (Figure 4d and Figure S22). Co‐expression involving CTSH and PDL1 emerged as a candidate pattern associated with early LC samples. EV‐centered analysis provided complementary evidence for LC‐associated vesicle subpopulations: *t*‐SNE visualization showed preferential expression of CTSH, PDL1, and MET in EVs from early LC patients compared with non‐cancer controls (Figure S23).

We further explored whether single‐EV findings could be summarized in a format compatible with conventional EV assays. Single‐EV profiling results were transformed into LC marker expression profiles of tetraspanin‐positive EVs (Figure 4e). In an exploratory receiver operating characteristic analysis comparing non‐cancer controls (*n* = 10) and early‐stage LC patients (*n* = 20), an EV signature comprising CTSH, PDL1, and MET showed the highest classification performance among the tested marker combinations (Figure 4f and Table S2), with an area under the curve of 0.93 (95% confidence interval, 0.85–1.00; DeLong's method [42]). Internal bootstrap and label‐permutation analyses further supported the robustness of this candidate marker combination within the pilot cohort (Methods). Together, these exploratory analyses illustrate how Cygnus‐guided single‐EV profiling can nominate marker combinations for future validation in ensemble EV assays. Because marker selection and evaluation were performed within the same pilot cohort, this apparent performance should be interpreted as exploratory and requires independent validation.

## 3 Discussion

Multiplexed single‐EV imaging provides a powerful approach for detecting tumor‐associated vesicle phenotypes that may be masked in bulk EV measurements. This capability is particularly important for early LC detection, where tumor‐derived EVs are expected to represent only a small fraction of the circulating vesicle population. In this study, we developed Cygnus, an end‐to‐end workflow that integrates cyclic immunofluorescence imaging with multiscale single‐EV analysis. Longitudinal plasma profiling in a genetically engineered mouse model revealed dynamic shifts in EV protein composition during tumor development and identified an EV subpopulation before tumors were detectable by radiography. Pilot analysis of human plasma further showed disease‐associated EV marker patterns in LC patients compared with non‐cancer controls, supporting the translational feasibility of single‐EV profiling for early LC biomarker discovery.

A central finding of this study is that marker co‐expression at the single‐vesicle level provides information that would be difficult to obtain from averaged EV measurements. Bulk assays can report increased expression of individual markers, but they cannot determine whether these markers arise from the same vesicles, separate vesicle populations, or nonspecific background. By preserving vesicle identity, multiplexed single‐EV imaging enabled us to identify combinatorial marker patterns and disease‐associated EV subpopulations. In the mouse model, EpCAM and CTSH co‐expression was detected in a week‐1 EV cluster and was supported by marker‐centered analysis. In clinical plasma, co‐expression patterns involving CTSH, PDL1, and MET provided candidate features for distinguishing LC‐associated EV profiles within the pilot cohort. These results suggest that early LC‐associated EVs may be better defined by combinatorial protein phenotypes than by single markers alone.

Cygnus was developed to support multiscale interpretation of these datasets. The workflow integrates preprocessing, marker co‐expression analysis, EV‐centered modeling, and sample‐level summarization. Its tapestry plots provide a global view of vesicle‐level marker distributions, while UpSet‐based analysis coupled with permutation testing identifies marker combinations that exceed expectations from random overlap. EV‐centered dimensionality reduction and clustering further resolve disease‐associated vesicle subpopulations. Finally, sample‐level summaries connect single‐EV phenotypes to ensemble EV readouts, providing a practical route for translating vesicle‐level discoveries into conventional assay formats. Although Cygnus was applied here to cyclic immunofluorescence imaging, the framework can accommodate multiplexed EV datasets generated by other tools (e.g., ImageJ, EVAnalyzer; Figure S24) and analytical platforms, such as nanoflow cytometry [43, 44, 45] and bead‐based EV assays [46, 47].

EVs are intrinsically multi‐class cargo carriers, simultaneously encapsulating proteins, nucleic acids, lipids, and structural information that reflects cellular state. Multiplexed EV characterization could therefore deepen our understanding of tumor biology under diverse physiological conditions, including tumor growth, treatment pressure, and metastatic progression. In future studies, integrating protein expression with additional EV features such as morphology, nucleic acid content, lipid composition, and vesicle size may provide more precise molecular snapshots of early tumor states. Such measurements could also inform diagnostic and intervention strategies by improving tumor detection and monitoring, supporting better‐powered trials, and guiding rational selection and timing of therapies.

This study has several limitations. First, the clinical cohort was designed as a pilot study to assess translational feasibility, and no independent validation cohort was included. Accordingly, the clinical analysis should be interpreted as an exploratory assessment of candidate EV signatures rather than definitive diagnostic validation. The reported performance of the CTSH/PDL1/MET combination may be affected by overfitting, as marker selection and evaluation were performed within the same cohort. Studies with larger independent cohorts are required to evaluate the robustness of the candidate EV signatures and establish their diagnostic performance. Second, the non‐cancer controls were younger than the LC patients, and the pilot cohort lacked clinically relevant high‐risk or disease‐control groups. Future studies should include age‐matched controls, high‐risk individuals (e.g., smokers), and patients with nonmalignant lung conditions (e.g., benign lung nodules or inflammatory lung disease) to assess the specificity and generalizability of the candidate EV signatures. Third, although vehicle controls were included in the mouse study, they do not fully rule out the effects of Ad‐Cre‐associated inflammation or tissue injury. Future studies using Ad‐Cre‐treated wild‐type mice or additional inflammation‐control models will be needed to evaluate these potential confounders.

Future work will also focus on expanding the Cygnus analytical workflow. Analytical robustness will need to be evaluated in broader experimental settings, including reproducibility across instruments, operators, sample types, staining panels, and analysis parameters. To facilitate external validation and community benchmarking, the Cygnus analysis package and ImageJ plugin have been made publicly available. Operationally, image processing could be automated by integrating neural network‐based segmentation tools (e.g., CellPose [48], ilastik [49]) directly within ImageJ workflows [50]. Developing an AI‐assisted agentic workflow could further help coordinate routine analysis steps, including quality control, parameter selection, report generation, and prioritization of marker combinations for validation. Additional statistical modules could support systematic comparison of EV molecular profiles across experimental conditions or treatment groups, analogous to differential expression analysis in single‐cell genomics. The exploratory CygnusVR module could further be extended by linking selected EV clusters to real‐time marker summaries, sample metadata, and biological annotations, enabling users to move more directly from visual inspection to interpretable EV phenotypes. Together, these extensions would strengthen multiplexed single‐EV imaging as a translational strategy for EV biomarker studies.

## 4 Methods

### 4.1 Cell Culture

Mouse lung adenocarcinoma cell line KP1.9 was established from lung tumors of C57BL/6 KP mice and was provided by Dr. A. Zippelius (University Hospital Basel, Switzerland). KP1.9 cells were cultured in Iscove's DMEM medium (Corning) supplemented with 10% fetal bovine serum (FBS, Bio‐Techne, S12450). Human lung adenocarcinoma cell lines, NCI‐H2228 (CRL‐5935) and A549 (CCL‐185), were purchased from ATCC (Manassas, VA). NCI‐H2228 and A549 cells were cultured in RPMI‐1640 (CellGro) and F‐12K (ATCC) medium, respectively, both supplemented with 10% FBS. All cell lines were maintained at 37°C in a humidified atmosphere with 5% CO_2_.

### 4.2 EV Preparation From Cell Culture

When cells reached 80%–90% confluence in culture, the medium was changed to the base medium with 5% exosome‐depleted FBS (Thermo, A2720803) for 48 hr. The conditioned medium was then collected and sequentially centrifuged at 300 × *g* for 5 min and 2,000 × *g* for 10 min to remove cellular debris. Subsequently, the supernatant was filtered through a membrane (0.22 µm pore size). The filtered supernatant was concentrated using a Centricon Plus‐70 device (Millipore Sigma) at 3,200 × *g* for 30 min. EVs were then isolated from the concentrated medium (0.5 mL) using a size‐exclusion column (SP1, Izon). Elution fractions F7‐F9 (1.5 mL each), containing the EVs, were collected into microcentrifuge tubes (Thermo, 90410). The isolated EVs were resuspended in 1× phosphate‐buffered saline (PBS) and stored at ‐80°C. EV concentrations were estimated by nanoparticle tracking analysis (NTA). For each sample, three 30‐sec video clips were acquired using a NanoSight LM10 (Malvern) and analyzed with NTA software (version 3.2).

### 4.3 Antibodies

Antibodies (Tables S3 and S4) used in this study were purchased and were conjugated to NHS‐Alexa Fluor dyes using Alexa Fluor 488/555/647 Labeling Kits per kit instructions (Thermo Scientific). The degree of labeling (DOL) was measured by a Nanodrop 1000 (Thermo Scientific). Labeled antibodies were stored in the dark at 4°C in PBS until use.

### 4.4 Multiplexed Single EV Imaging

For universal EV identification, EVs were labeled with the NHS‐ester form of Alexa Fluor 555, which reacts with amine groups on the EV surface. A 300 ng (3 µL) aliquot of EVs was combined with 2 µL of 0.1 M sodium bicarbonate buffer (pH 8.5) and 0.2 µL of Alexa Fluor 555 NHS ester (10 mg/mL in DMSO). After 1 hr incubation at 20°C, excess dye was removed via two sequential purifications using 40K Zeba Spin Desalting Columns (Thermo, 87765). Subsequently, 50 ng (5 µL) of Alexa Fluor 555‐labeled EVs were captured on a poly‐L‐lysine‐coated glass slide (Electron Microscopy Science, 63429‐04) by incubation for 30 min (20°C). Captured EVs were fixed with 4% paraformaldehyde for 15 min. Following three washes with PBS, EVs were blocked for 30 min with SuperBlock (Thermo Scientific, 37535). Fluorescently labeled antibodies were then incubated on the slide for 1 hr (20°C). The slide was subsequently washed three times with PBS and sealed with a coverslip. Fluorescent images were acquired using an upright fluorescent microscope (Olympus, BX63) equipped with a 40× objective, with a 500‐msec exposure time for each channel (FITC, Texas Red, and Cy5). Following initial imaging, the slide was incubated with a quenching buffer (3% v/v H_2_O_2_) for 30 min and washed with PBS. EVs were then imaged again to assess background signals. This cycle of quenching and imaging was repeated to label EVs with additional markers.

### 4.5 Image Alignment and EV Detection

A custom‐designed Fiji plugin [51] (Note S1) was used to correct positional shifts (translation and rotation) introduced during manual imaging procedures. The alignment algorithm operated under two assumptions: i) each image contained a minimum of two identifiable fluorescence reference points, and ii) positional variations were restricted to translational and rotational displacements without scaling artifacts. The alignment procedure involved three steps: first, two distinct fluorescent points were manually identified and annotated in each image to serve as fiducial markers; second, a reference line was established between these markers to define the spatial and angular relationship between images; and third, images were computationally aligned by superimposing these reference lines. The process led to the precise superposition of fluorescence signals from individual EVs across multiple images. The aligned image datasets were subsequently analyzed using AI‐powered image analysis software (AIVIA) for EV detection and segmentation. For each detected EV, fluorescence intensities for each marker were quantified.

### 4.6 LC Marker Selection

Candidate LC‐associated EV protein markers were selected using a combination of transcriptomic analysis, protein localization filtering, EV annotation, literature review, and experimental prioritization. Public RNA sequencing data from *Kras* ^G12D/+^; *Trp53* ^−/−^ (KP) lung tumors at early and late stages were used to identify genes overexpressed in early LC compared with normal lung tissue [32]. FPKM values from two normal lung samples and six early tumor samples were row‐wise z‐transformed for each gene. We calculated *z_t_* as the average z‐score across early tumor samples and *z_n_* as the average z‐score across normal lung samples. Genes with *z_t_* − *z_n_* > 2 were defined as early tumor‐overexpressed candidates, yielding 509 genes from 24,211 screened genes. These candidates were filtered for plasma‐membrane and transmembrane protein localization, excluding mitochondrial membrane proteins, resulting in 69 genes. Vesiclepedia annotations were then used to identify proteins with reported EV associations, yielding 67 candidate markers.

From the top 20 candidates ranked by *z_t_* − *z_n_* score (CD63, SDC1, MET, BMPR1A, SLC34A2, CTSH, TMED2, ATP1B1, SPINT2, AQP5, ALCAM, SLC12A2, MUC1, CDH1, CD320, PTPRF, CLDN3, ZDHHC3, EPCAM, SPINT1), five protein markers were selected based on LC relevance and antibody availability: SDC1, MET, CTSH, MUC1, and EpCAM. We added additional EGFR, PDL1, and HER2 markers because of their clinical relevance as LC‐associated therapeutic or immuno‐oncology markers [52]. ADAM10 and SP‐B were included based on prior work related to LC detection [36] and lung‐organ specificity [37], respectively. For clinical plasma profiling, we prioritized markers that showed tumor‐associated temporal increases in the longitudinal mouse study. This criterion led to the selection of EpCAM, CTSH, SDC1, MET, EGFR, and PDL1 for the final clinical panel.

### 4.7 Cygnus Package Development

The Cygnus package [53] was implemented in R version 4.4.1. The package integrated multiple R libraries: ggplot2, plotly, and ComplexUpset for data visualization; uwot, Rtsne, leiden, igraph, and dbscan for dimensionality reduction and clustering analyses; and tidyverse for data manipulation. The package followed an S4 object‐oriented architecture. The core class, CygnusObject, contained five slots: matrices (intensity data), ev_meta (sample metadata), markers_meta (marker annotations), marker_analysis (marker analysis results), and dim_red (dimensionality reduction outputs). Comprehensive documentation and tutorials were available through vignettes, and the package website is built with pkgdown (version 2.1.3).

### 4.8 Cygnus Analysis Workflow

Single‐EV intensity tables were imported into Cygnus and stored as CygnusObject objects together with sample and marker metadata. Marker‐specific thresholds were defined using control IgG measurements to convert continuous intensity values into binary marker‐positive or marker‐negative calls. Processed data were visualized as tapestry plots to display marker‐presence patterns across individual EVs. Marker‐centered analyses included expression frequency, pairwise correlation, and UpSet‐based enumeration of marker combinations. EV‐centered analyses used marker intensity profiles for dimensionality reduction and clustering, including PCA, *t*‐SNE, UMAP, KNN, Leiden, and HDBSCAN methods. Sample‐level summaries were generated by aggregating vesicle‐level measurements into marker expression profiles of defined EV populations, including tetraspanin‐positive EVs.

### 4.9 Permutation Analysis

To assess whether observed co‐localization patterns exceeded expectations from random coincidence, we implemented a permutation‐based statistical test in the Cygnus R package. Let M denote the set of all profiled markers. For each marker *k* ∈ M, its expression frequency *p*_k_ was defined as *p*_k_ = *n*_k_/*n*, where *n*_k_ was the observed number of EVs positive for marker *k*, and *n* was the total number of EVs. Under the *null hypothesis* that marker expressions were independent across EVs, the expected number of EVs (*n*_C_) expressing a specific marker subset C ⊆ M was calculated as

$$\;n_{C}=n\cdot \prod_{k\in C}p_{k}\cdot \prod_{k\in \left(M-C\right)}\left(1-p_{k}\right).$$

The deviation (*d*_C_ = *n*_C0_ – *n*_C_) between the observed EV count (*n*_C0_) and the expected count (*n*_C_ from the null hypothesis) then served as the test statistic. We performed a permutation test by randomly assigning each marker label (*k*) to the *n* EVs while preserving its observed expression frequency *p*_k_. Repeating this procedure generated a null distribution of *d*_C_ under marker independence, from which *p*‐values were computed [31]. Bonferroni correction was applied to account for multiple testing across evaluated marker subsets.

### 4.10 Profiling Single EVs From Lung Cancer Mice

All animal experiments were conducted in accordance with protocols approved by the Institutional Animal Care and Use Committee of Massachusetts General Hospital (protocol 2019N000133). 129S1 mice harboring Cre‐activatable conditional *Kras* ^LSL‐G12D/^; *Trp53* ^flox/flox^ alleles were used as an autochthonous model of non‐small cell lung cancer [38]. Tumor induction was achieved by intratracheal administration of 3 × 10^7^ adenovirus particles expressing Cre recombinase (Ad‐Cre; University of Iowa Viral Vector Core). Following Ad‐Cre administration, 100 µL of blood was collected weekly via retro‐orbital bleeding into EDTA‐containing tubes. Terminal blood collection (≈1 mL) was performed by cardiac puncture upon reaching advanced disease endpoints. Plasma was isolated by centrifugation at 1,500 × *g* for 15 min at 4°C and stored at ‐80°C until use. For EV isolation, we used size‐exclusion chromatography (SEC) columns (qEV SP2, Izon), which were compatible with volume‐limited longitudinal samples. A 40 µL plasma aliquot was diluted to 60 µL in PBS and processed using the SEC column. All mouse samples were processed by the same SEC workflow to ensure consistent comparisons of the longitudinal data. Obtained EVs were processed for imaging as described above.

### 4.11 Tumor Imaging in Mice

Micro‐computed tomography (µCT) imaging was performed on mice subsequent to blood collection. Tumor‐free cage‐mates were included as controls. High‐resolution µCT scans were acquired using an Inveon system (Siemens) with the following parameters: 80 kVp beam energy, 500 µA current, and 425 msec exposure time over 360 projections. Images were reconstructed using a modified Feldkamp cone‐beam reconstruction algorithm (Cobra, Exxim Inc.). Tumor burden in the lung was assessed with direct visualization and subsequently quantified using publicly available software implemented in MATLAB [54]. Specifically, the thorax was manually segmented from reconstructed whole‐body µCT images and processed using the MLAST pipeline. A visual inspection and manual refinement were performed to exclude extraneous tissues from the mediastinum, diaphragm, and surrounding chest wall prior to quantification. Discrete disease at each time point was visually assessed and tabulated. Furthermore, both discrete and confluent disease volumes were calculated using the MLAST pipeline, and fractional tumor burden was determined relative to the total lung volume.

### 4.12 EV Profiling With Clinical Samples

This study was approved by the Institutional Review Boards of Massachusetts General Hospital (protocols 2017P001581, 2019P003441, 2019P003472; principal investigator: H.L.) and Chonnam National University Hwasun Hospital (CNUHH 2019–228; principal investigator: C.H.K.). All procedures were conducted in accordance with institutional guidelines, and written informed consent was obtained from all participants prior to enrollment. Peripheral blood samples (≈10 mL) were collected from study participants and centrifuged at 2,000 × *g* for 20 min to separate plasma from cellular components. Plasma samples were stored at ‐80°C until use. For EV isolation, stored plasma samples (500 µL) were thawed and purified using dual‐mode chromatography (DMC) according to established protocols [39]. All clinical samples were processed using the same DMC workflow; therefore, group‐wise comparisons were internally consistent. Obtained EVs were processed for imaging as described above.

### 4.13 Clinical ROC Analysis

For clinical plasma analysis, vesicle‐level measurements were summarized as LC marker expression profiles in tetraspanin‐positive EVs. Receiver operating characteristic (ROC) curves were generated to evaluate the exploratory classification performance of individual markers and marker combinations within the pilot clinical cohort. For marker combinations, logistic regression models were fitted using the selected markers, and predicted probabilities were used for ROC analysis. Area under the curve (AUC) values and 95% confidence intervals were calculated using pROC. Marker combinations were compared to identify the minimal panel with the highest AUC in this cohort.

### 4.14 Internal Robustness Analyses

To assess the internal robustness of the CTSH/PDL1/MET combination, we performed stratified bootstrap and label‐permutation analyses. For the stratified bootstrap analysis, non‐cancer control and early LC samples were resampled within each group, and the CTSH/PDL1/MET logistic panel was re‐evaluated in 5,000 resampled datasets. The panel retained high performance across resamples, with a median AUC of 0.945 and AUC ≥ 0.90 in 79.6% of bootstrap resamples. For label‐permutation testing, class labels were randomly permuted 10,000 times while preserving the number of control and early LC samples, and the observed CTSH/PDL1/MET AUC was compared with the null distribution obtained under random label assignment. The observed AUC of 0.93 was unlikely under random class labels (*p* = 0.0009).

### 4.15 CygnusVR Development

The CygnusVR application [55] was developed using the Unity 3D game engine with C# scripting, selected for its extensive VR development resources (e.g., the “Create with VR” course on Unity Learn) and its active developer community. The application features two analytical interfaces: i) an Immersive View enabling simultaneous analysis and comparison of multiple 2D results through an interactive swipe menu interface, and ii) a Clustering Scene for loading and manipulating 3D clustering data with selective cluster visualization capabilities. GPU instancing and dynamic batching techniques were implemented to maintain real‐time performance (>30 frames per second) during 3D data interactions. Application development was conducted on a Windows 10 workstation equipped with an Intel i7‐7820HK processor, 16GB RAM, and an NVIDIA GTX 1070 graphics card. The VR operation utilized a Meta Quest 2 standalone headset (6GB RAM, 1832×1920 per eye resolution, 72–120 Hz refresh rate) paired with two haptic‐enabled controllers. Wireless communication was established between the headset and the workstation for software installation.

## Author Contributions

**M.H.C**., **M.A.M**., and **H.L**. conceptualized and designed the study. **M.H.C**. conducted the experiments and performed data analysis. **Yein C**. and **Yoonjeong C**. developed the Cygnus data analysis and VR modules, respectively. **B.C**. developed the Fiji plugin. **M.H.C**., **N.O**., **H.L**., and **M.A.M**. designed and analyzed the animal model studies. **T.S.C.N**. analyzed the CT data obtained from murine imaging. **J.S**., **A.J**., **H.K**., and **H.‐K.W**. provided technical assistance with single EV imaging. **C.‐H.K**. collected blood samples from LC patients and analyzed their clinical data. **C.M.C**. analyzed clinical data. **M.H.C**., **Yein C**., **Yoonjeong C**., **M.A.M**., and **H.L**. prepared the figures and drafted the manuscript, with contributions from all authors.

## Conflicts of Interest

The authors declare no conflicts of interest. M.A.M. has received research funding from Ionis Pharmaceuticals and non‐financial support from Genentech/Roche, Pfizer, and Archon Biosciences, all outside the submitted work.

## Supporting information




**Supporting File 1**: advs77301‐sup‐0001‐SuppMat.pdf.

**Supporting File 2**: advs77301‐sup‐0002‐MovieS1.mov.

**Supporting File 3**: advs77301‐sup‐0003‐MovieS2.mov.

## Data Availability

The Custom ImageJ Plugin for Multi‐image Alignment, Cygnus Analysis Package, and CygnusVR Software Are Publicly Available on GitHub at Https://Github.com/kylie0914/Multi‐Image‐Alignment‐Code, Https://Github.com/Yeinchung/Cygnus, and Https://Github.com/kylie0914/CygnusVR_Unity, Respectively. The Versions Associated With this Study Are Archived on Zenodo [51, 53, 55].
