# Supplementary material for: Multiplexed Profiling of Individual Extracellular Vesicles Reveals Lung Cancer‐Associated Protein Signatures
Source: Adv Sci (Weinh). 2026 Aug 25:e77301. Online ahead of print. doi: 10.1002/advs.77301 (PMC13505464; doi:10.1002/advs.77301)
Supplement: Supplementary file 1 [file ADVS-9999-e77301-s002.pdf]

SUPPLEMENTARY INFORMATION

Multiplexed profiling of individual extracellular vesicles reveals lung cancer-associated protein signatures

Mi Hyeon Cho[1,2,3†], Yein Chung[1,4†], Yoonjeong Choi[1,2†], Baekdong Cha[5], Jayeon Song[1,2], Nuri Oh[1,2], Ala Jo[1,2,6,7], Thomas S. C. Ng[1,2], Hyunho Kim[1,2], Hyun-Kyung Woo[1,2], Chang Hyun Kim[8], Cesar M. Castro[1,9], Miles A. Miller[1,2*], and Hakho Lee[1,2*]

[1] Center for Systems Biology, Massachusetts General Hospital Research Institute, Boston, MA, USA
[2] Department of Radiology, Massachusetts General Hospital, Harvard Medical School, Boston, MA, USA
[3] Metropolitan Seoul Center, Korea Basic Science Institute (KBSI), Seoul, Republic of Korea
[4] Department of Immunology, University of Toronto, Toronto, ON, Canada
[5] Department of Industrial Machinery DX., Korea Institute of Machinery & Materials (KIMM), Daejeon, Republic of Korea
[6] Department of Physiology, College of Medicine, Ewha Womans University, Seoul, Republic of Korea
[7] Inflammation-Cancer Microenvironment Research Center, College of Medicine, Ewha Womans University, Seoul, Republic of Korea
[8] Department of Surgery, Chonnam National University Hwasun Hospital and Medical School, Jeonnam, Republic of Korea
[9] Department of Medicine, Massachusetts General Hospital, Harvard Medical School, Boston, MA, USA

†These authors contributed equally.

Corresponding Authors:

Miles A. Miller, PhD | (+1)-617-643-0500 | miles.miller@mgh.harvard.edu

Hakho Lee, PhD | (+1)-617-726-6487 | hlee@mgh.harvard.edu

Table of Contents

Fig. S1. EV retention across cyclic imaging rounds 3
Fig. S2. Single-fluorophore bleed-through analysis 4
Fig. S3. Background and detergent controls for Cygnus EV imaging 5
Fig. S4. EV dilution analysis 6
Fig. S5. Nearest-neighbor (NN) distance analysis of detected EV-associated spots 7
Fig. S6. Assessment of cycle-dependent staining bias for LC-associated markers 8
Fig. S7. Estimation of false co-localization by mixing pre-labeled EV preparations 9
Fig. S8. EV profiling of H2228 cell line-derived EVs 10
Fig. S9. EV profiling of A549 cell line-derived EVs 11
Fig. S10. Reproducibility of EV identification 12
Fig. S11. Limitation of traditional Venn diagrams 13
Fig. S12. Cygnus VR display 14
Fig. S13. Marker selection for early LC detection 15
Fig. S14. $Kras^{G12D}$ immunohistochemistry during lung tumor initiation and progression in the KP mouse model 16
Fig. S15. µCT images of tumor-bearing mice 17
Fig. S16. Upset plot analysis of EVs before tumor induction 18
Fig. S17. Distinct EV profiles in week 1 of LC development 19
Fig. S18. Longitudinal EpCAM- and CTSH-positive EV fractions in individual mice 20
Fig. S19. Immunohistochemical (IHC) staining of tumor tissue 21
Fig. S20. Characterization of DMC-processed plasma EV preparations 22
Fig. S21. Profiling of plasma EVs from clinical samples 23
Fig. S22. UpSet and permutation analysis of marker co-expression in a stage IV LC plasma sample ..24
Fig. S23. Single-EV population structure in early-stage lung cancer 25
Fig. S24. Cygnus application with other EV-image analysis tools 26
Table S1. Clinical information of cohorts involved in LC detection 27
Table S2. Classification performance of EV marker combinations for distinguishing non-cancer controls from stage I/II lung cancer 28
Table S3. Antibodies for mouse plasma-derived EVs 29
Table S4. Antibodies for human cell line-derived EVs 30
Supporting Movie Legends 31
Supplementary Note: Multi-image Alignment Code 32

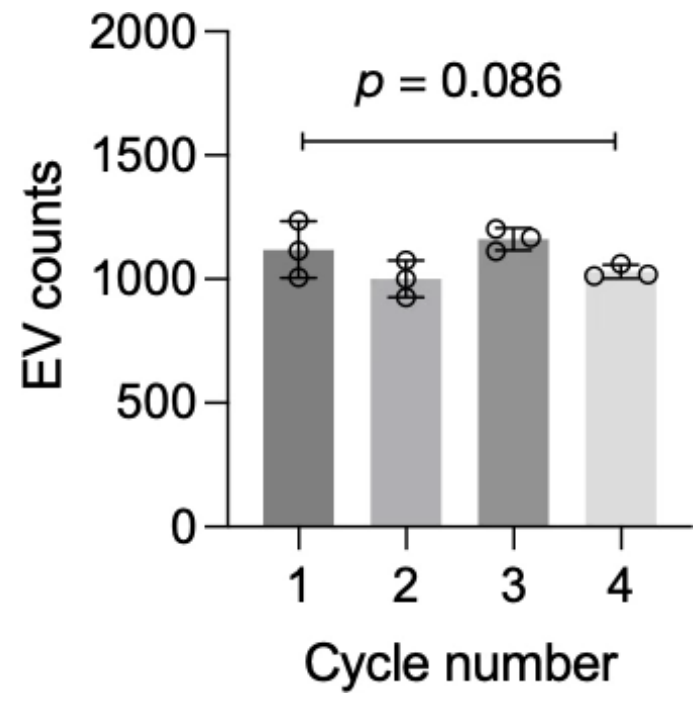

Fig. S1. EV retention across cyclic imaging rounds. Pan-EV-positive particle counts were quantified across imaging cycles. EV counts remained largely stable across cycles ($p = 0.086$; one-way ANOVA). The normalized mean counts were 89.6%, 104.8%, and 92.9% relative to cycle 1 for cycles 2–4, respectively. Data are mean ± s.d. from technical triplicates.

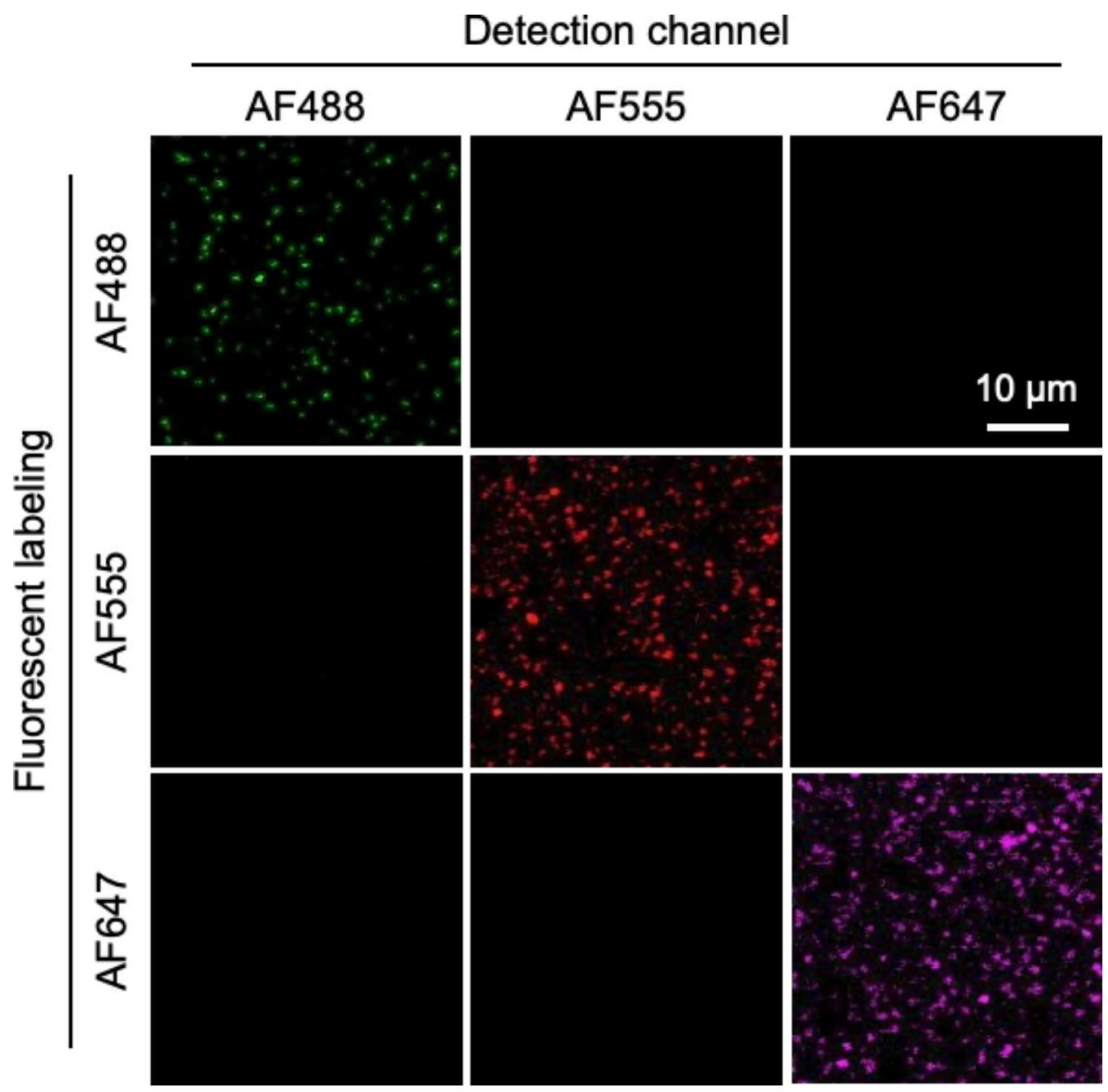

Fig. S2. Single-fluorophore bleed-through analysis. Alexa Fluor (AF) 488, AF555, and AF647 fluorophores were assessed under Cygnus imaging conditions. In a 3 × 3 fluorophore/channel matrix, EV signals were detected only in the matched dye-channel pairs, with negligible off-channel signal.

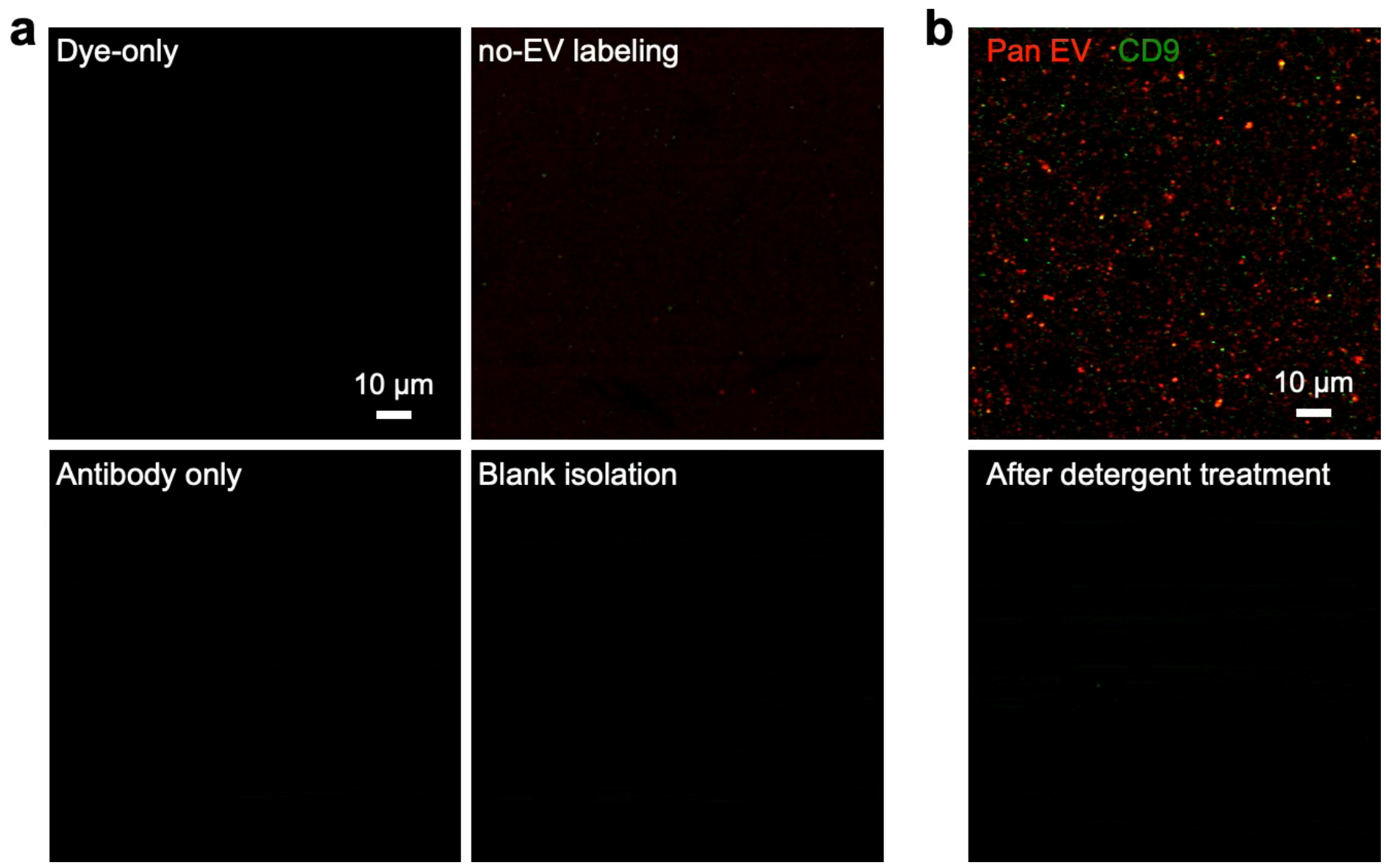

Fig. S3. Background and detergent controls for Cygnus EV imaging. (a) Dye-only, no-EV labeling, antibody-only, and blank-isolation controls produced few detected spots, indicating minimal background from staining or EV-isolation procedures. **(b)** Detergent sensitivity test. KP1.9-derived EVs were stained with NHS-Alexa Fluor total protein dye (pan-EV, red) and anti-CD9 antibody (green), then imaged before and after treatment with 1% Triton X-100. Detergent treatment eliminated fluorescent particle signals, supporting that the detected signals originated from membrane-associated EV-like particles.

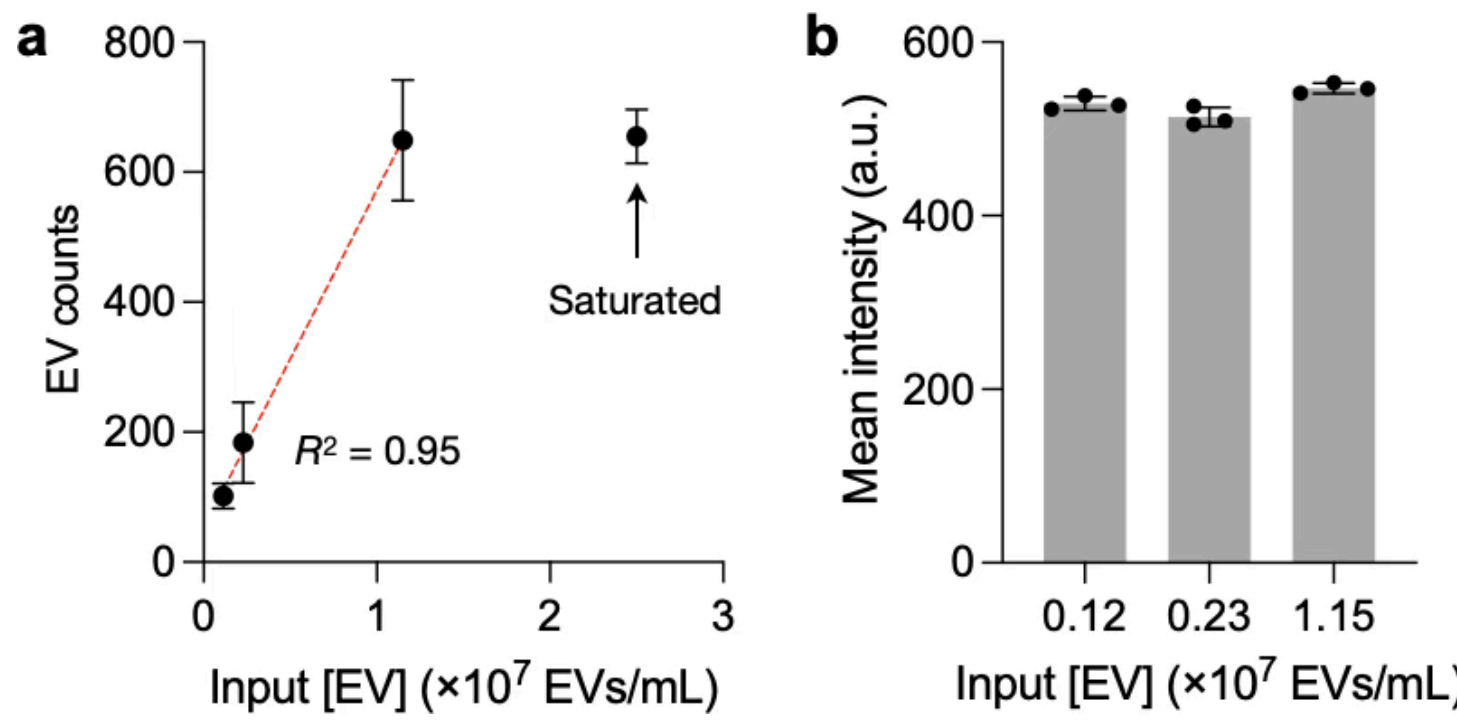

Fig. S4. EV dilution analysis. KP1.9-derived EVs were serially diluted, stained with NHS-Alexa Fluor total protein dye, and imaged by Cygnus. **(a)** Counts of pan-EV-positive particles increased with EV input concentration at lower concentrations ($R^2 = 0.95$), while a higher input concentration showed signal saturation. **(b)** Mean fluorescence intensity of detected pan-EV-positive particles remained largely unchanged across EV input concentrations. Data are mean ± s.d. from technical replicates ($n = 3$).

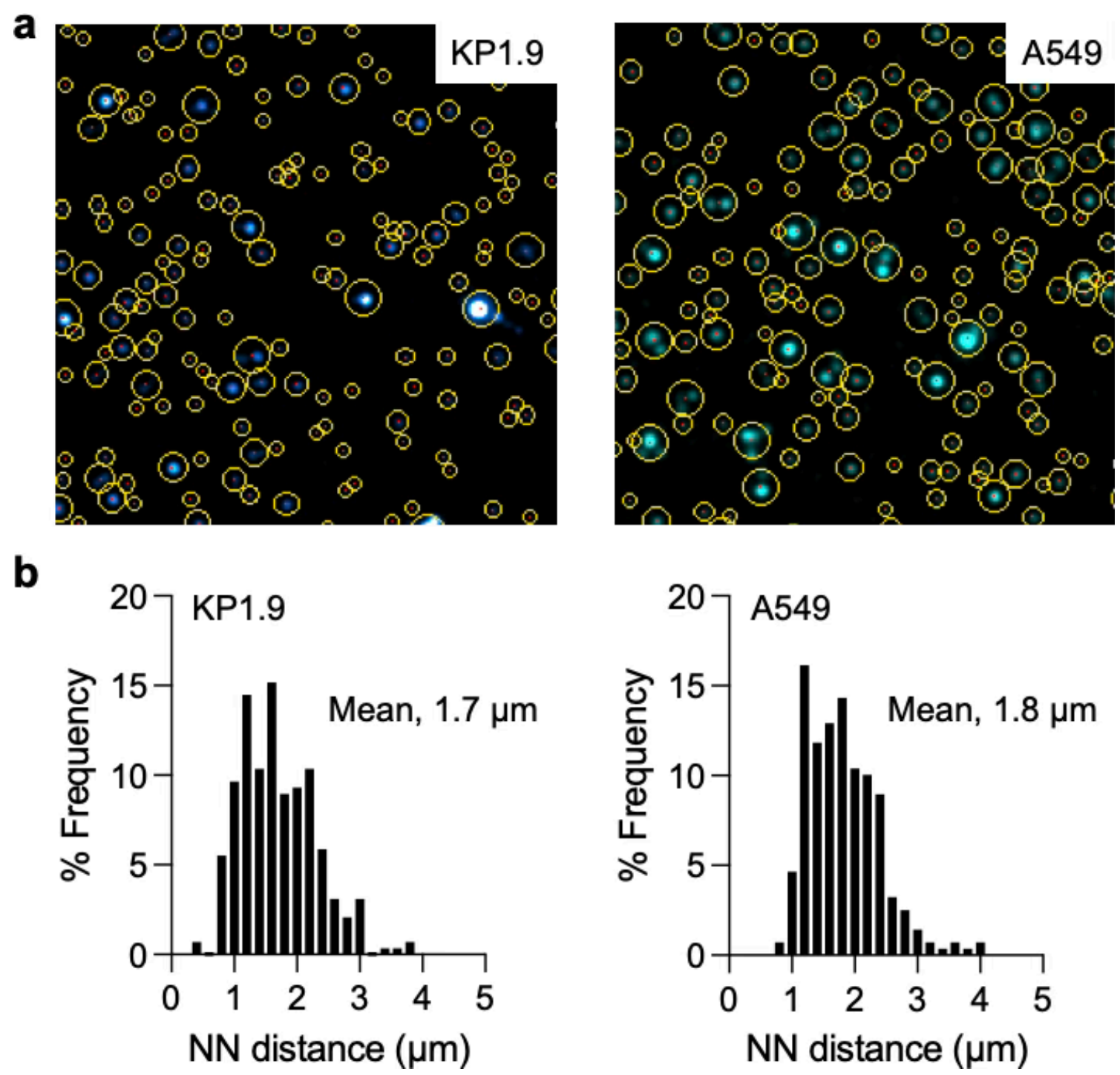

Fig. S5. Nearest-neighbor (NN) distance analysis of detected EV-associated spots. (a) Representative Cygnus images of KP1.9- and A549-derived EVs (outlined in yellow). **(b)** Histograms of NN distances between detected spots. The mean NN distances were 1.7 *µ*m (KP1.9) and 1.8 *µ*m (A549). More than 99% of detected spots were separated by distances greater than the microscope's diffraction limit (~0.6 *µ*m).

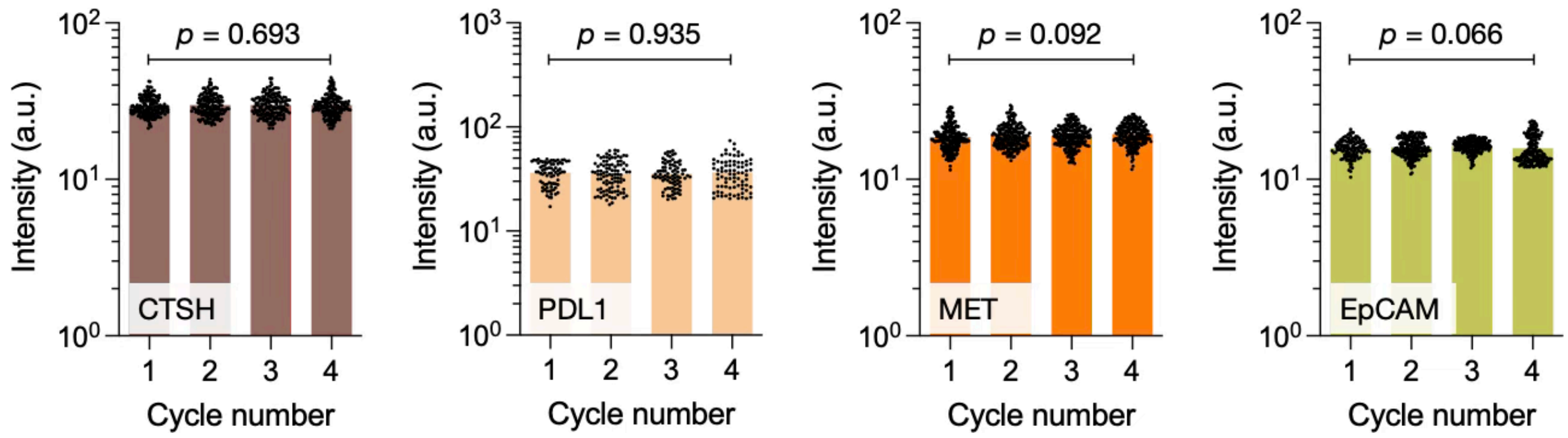

Fig. S6. Assessment of cycle-dependent staining bias for LC-associated markers. EV aliquots were stained for CTSH, PDL1, MET, or EpCAM at different imaging cycles after repeated quenching steps, and fluorescence intensities were measured. Mean fluorescence intensities showed no significant dependence on cycle number for CTSH ($p = 0.693$), PDL1 ($p = 0.935$), MET ($p = 0.092$), and EpCAM ($p = 0.066$) by one-way ANOVA.

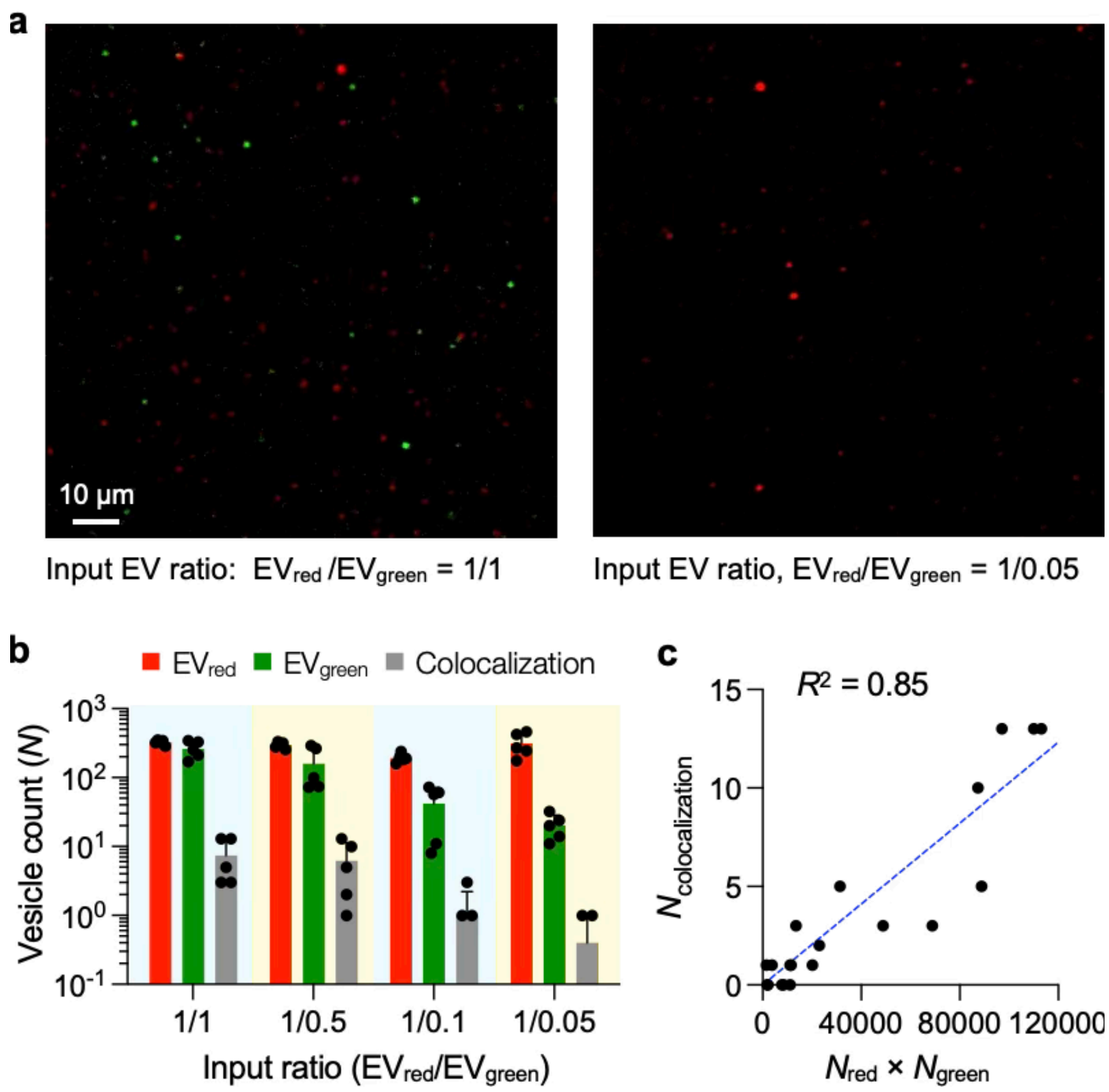

Fig. S7. Estimation of false co-localization by mixing pre-labeled EV preparations. (a) Representative fluorescence images of EV preparations separately labeled with red or green pan-EV dyes and mixed at the indicated EV_{red}/EV_{green} ratios before Cygnus imaging. **(b)** Counts of red EVs, green EVs, and red/green double-positive spots across the tested mixing ratios. The red/green double-positive spots represent false co-localization events caused by random spatial overlap. Data are from five technical replicates. **(c)** False co-localization counts scaled with the product of red and green EV counts ($N_{red} \times N_{green}$), consistent with density-dependent random overlap.

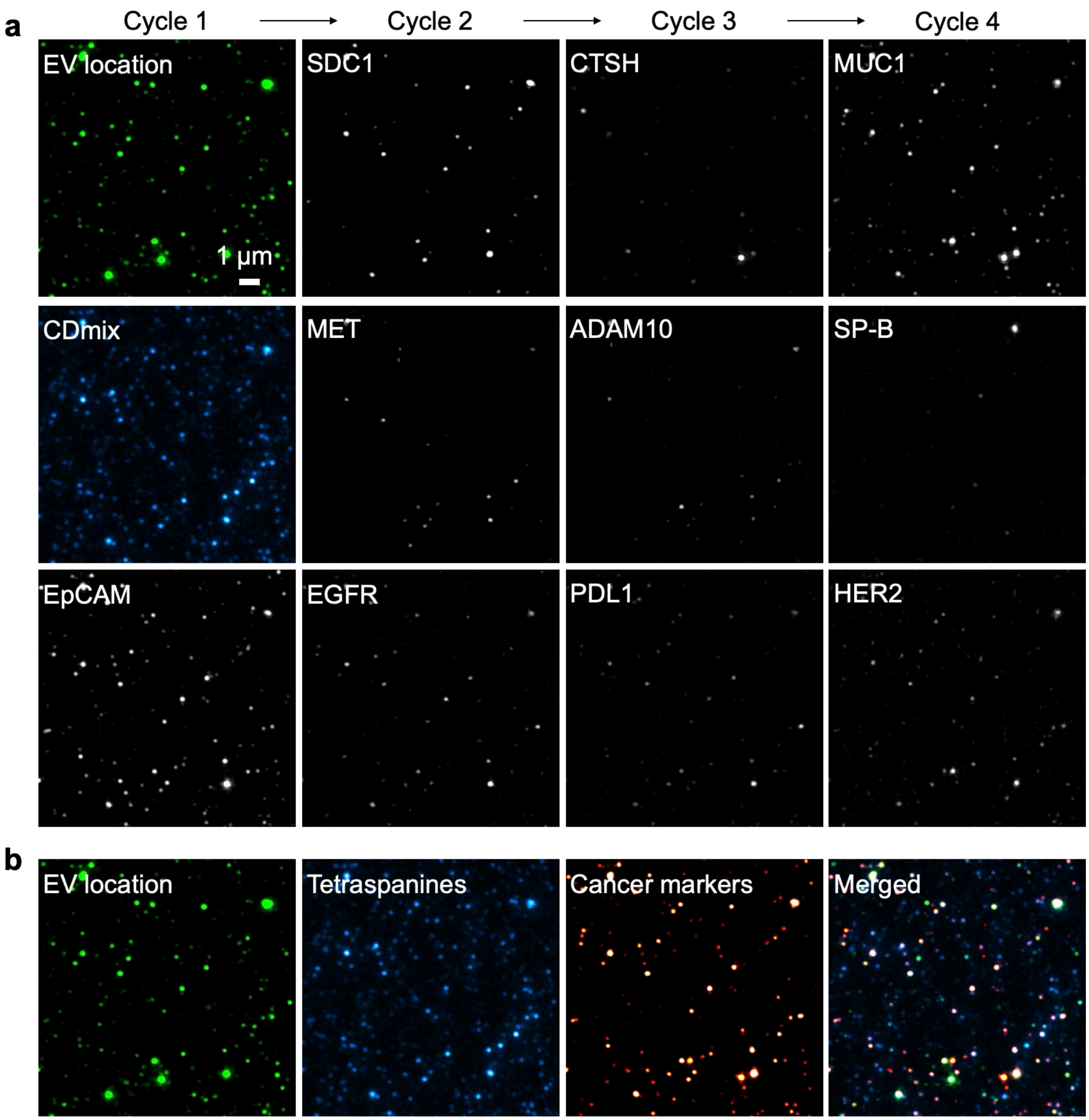

Fig. S8. EV profiling of H2228 cell line-derived EVs. (a) Measurement of 11 markers on EVs through multiplexed single EV imaging. For universal EV identification (Pan-EV), amine groups on the EV surface were labeled with the NHS-ester form of a fluorescent dye. A mixture of CD9, CD63, and CD81 antibodies (referred to as CD_{mix}) was used to identify tetraspanin-expressing EVs. Nine cancer markers and one lung-specific marker (i.e., SP-B) were analyzed. **(b)** Merged image of Pan-EV, Tetraspanins, and 10 cancer marker images.

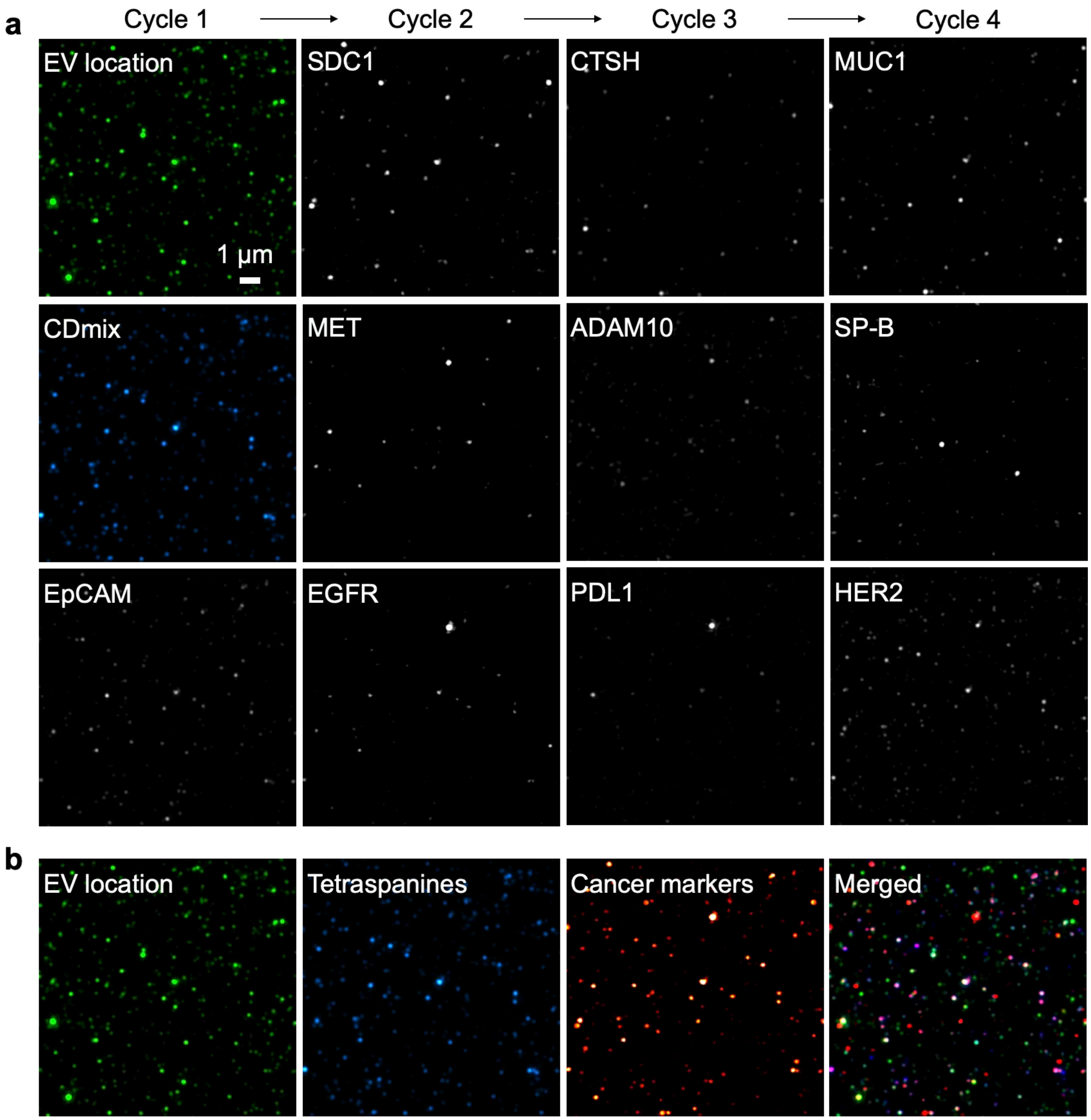

Fig. S9. EV profiling of A549 cell line-derived EVs. (a) Measurement of 11 markers on EVs through multiplexed single EV imaging. For universal EV identification (Pan-EV), amine groups on the EV surface were labeled with the NHS-ester form of a fluorescent dye. A mixture of CD9, CD63, and CD81 antibodies (referred to as CD_{mix}) was used to identify tetraspanin-expressing EVs. Nine cancer markers and one lung-specific marker (i.e., SP-B) were analyzed. **(b)** Merged image of Pan-EV, Tetraspanins, and 10 cancer marker images.

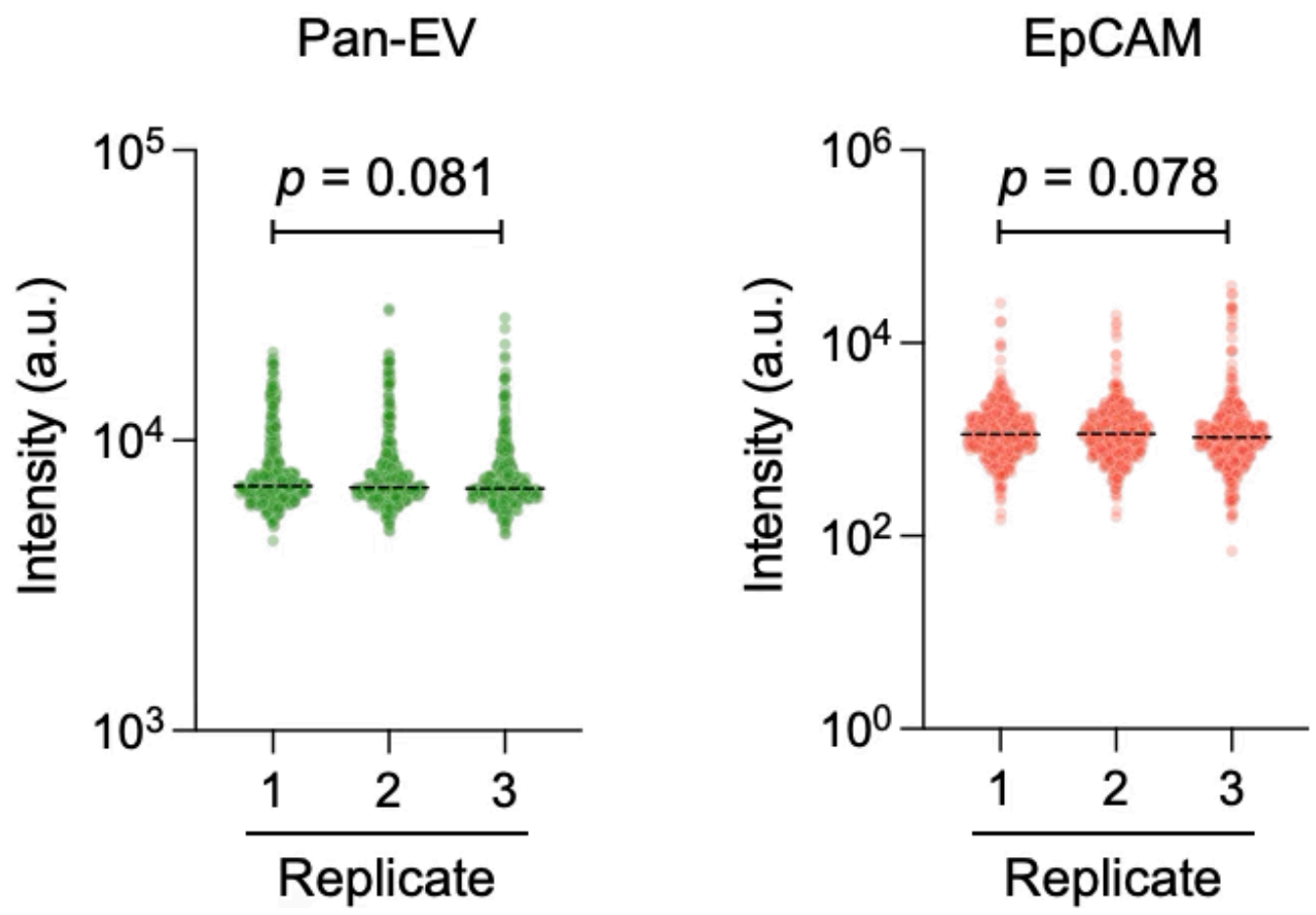

Fig. S10. Reproducibility of EV identification. The reproducibility of the EV detection algorithm (trained in AIVIA) was assessed. A single-parent EV sample was aliquoted into three replicates, and each aliquot was subjected to single EV imaging. Image analysis identified EVs based on a pan-EV signal and quantified EpCAM expression. No statistically significant differences ($p > 0.05$; one-way ANOVA) were observed in the measured signal intensities across the three replicates. Five hundred EVs were analyzed per sample. Dotted lines indicate median values.

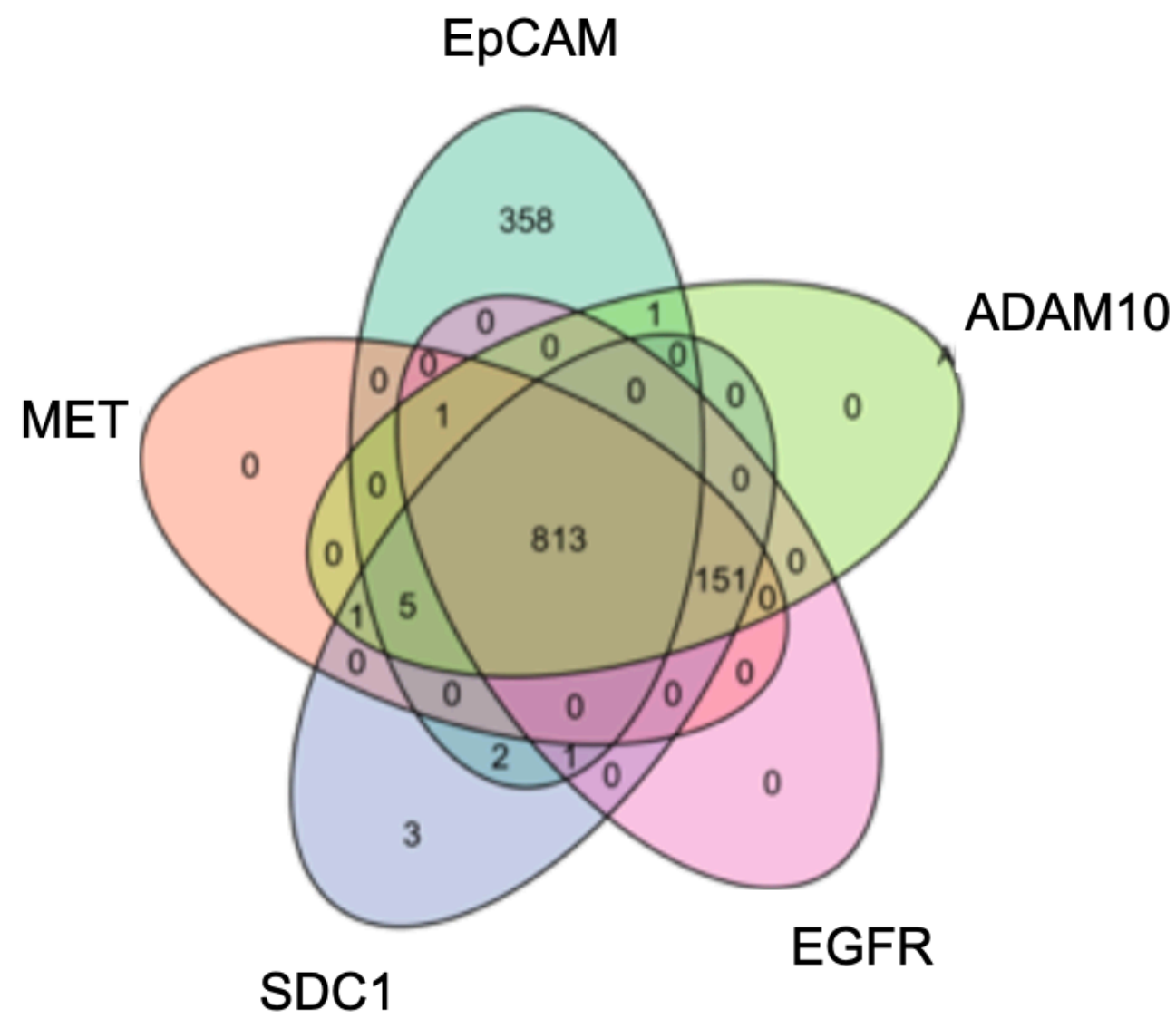

Fig. S11. Limitation of traditional Venn diagrams. Co-localization of protein markers was visualized using single-particle imaging data from KP1.9 EVs. Traditional Venn diagrams become difficult to construct and interpret when applied to datasets with many intersecting sets.

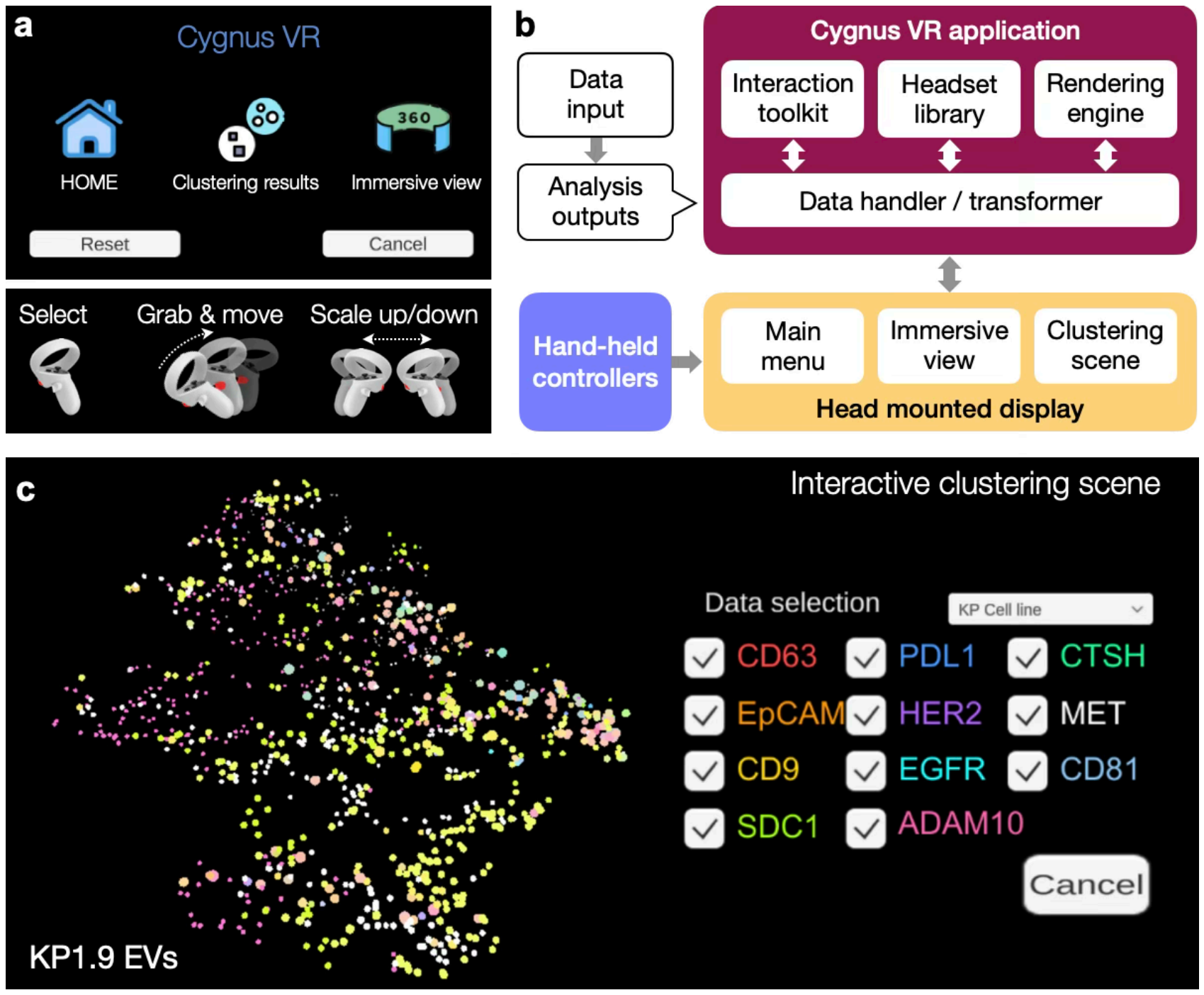

Fig. S12. Cygnus VR display. (a) The CygnusVR module presents a user interface accessible via a head-mounted display (HMD) and handheld controllers, enabling intuitive gesture-based interactions with Cygnus results. **(b)** Architecture of the CygnusVR system. The VR application has three core components: i) an interaction toolkit processing user gestures (e.g., grab, move, and scale), ii) a headset library managing head tracking and hand controller inputs, and iii) a rendering engine delivering real-time 3D visualization. This software runs as a standalone HMD application. **(c)** An example of the Cygnus VR display. In the 3D clustering scene, users can select marker-specific EVs and spatially manipulate clusters via rotation, zooming, and scaling. This capability helps identify EV subpopulations that are subtle or occluded in conventional 2D representations. The displayed *t*-SNE plot was generated from KP1.9 EV profiling results.

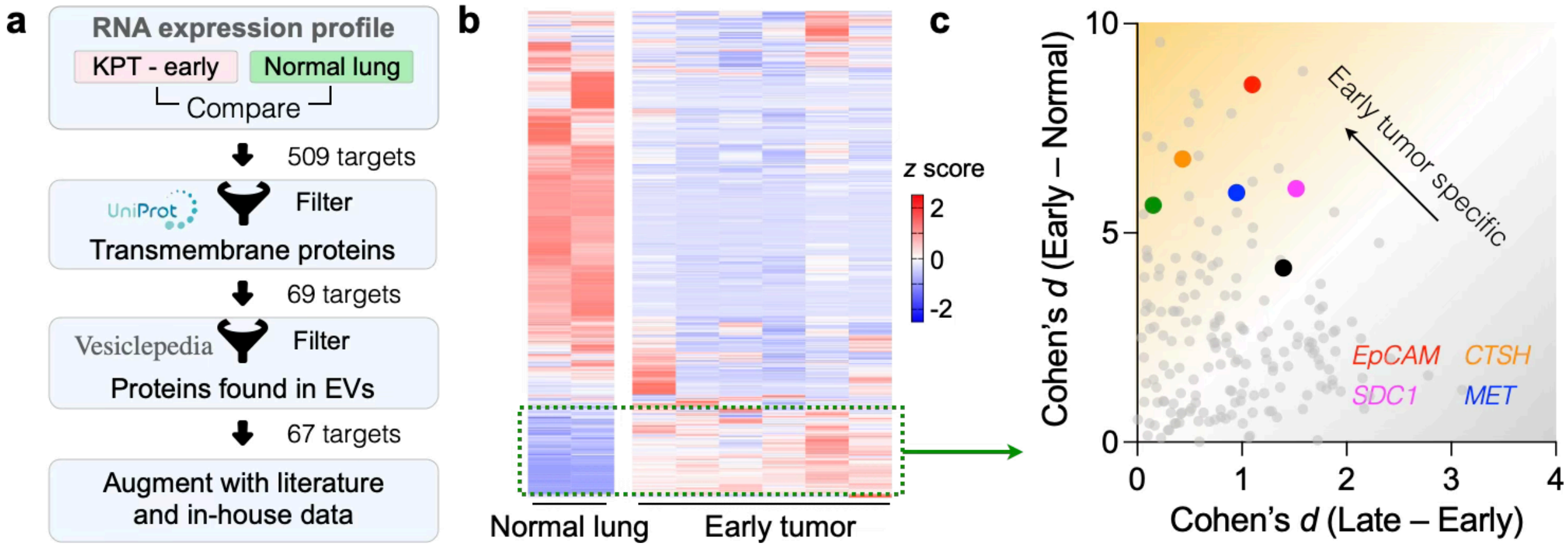

Fig. S13. Marker selection for early LC detection. (a) Selection algorithm. Public databases were mined to determine EV surface proteins that could be overexpressed in early LC. **(b)** Heatmap of proteins found in EVs from the normal lung and early tumor. **(c)** The algorithm selected candidate markers (e.g., EpCAM, CTSH, SDC1, MET) for early LC detection.

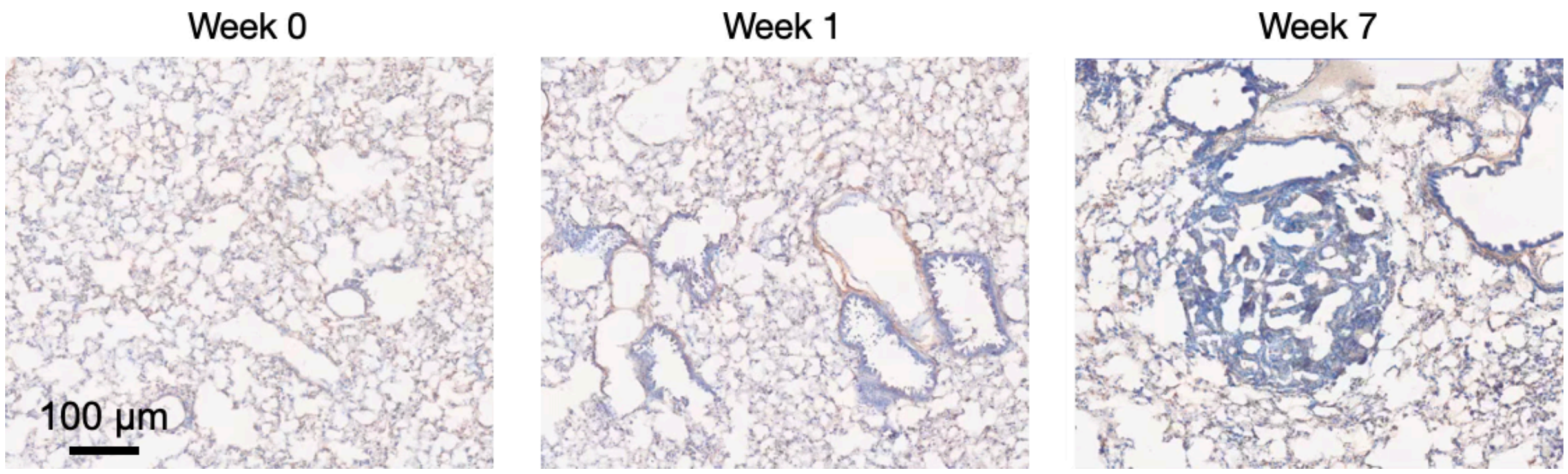

Fig. S14. $Kras^{G12D}$ immunohistochemistry during lung tumor initiation and progression in the KP mouse model. Representative lung sections stained for $Kras^{G12D}$ before tumor induction (week 0) and at weeks 1 and 7 after intratracheal Ad-Cre administration. $Kras^{G12D}$-positive epithelial foci were detectable at week 1 and became more prominent by week 7, consistent with progressive expansion of oncogene-initiated lung epithelial lesions.

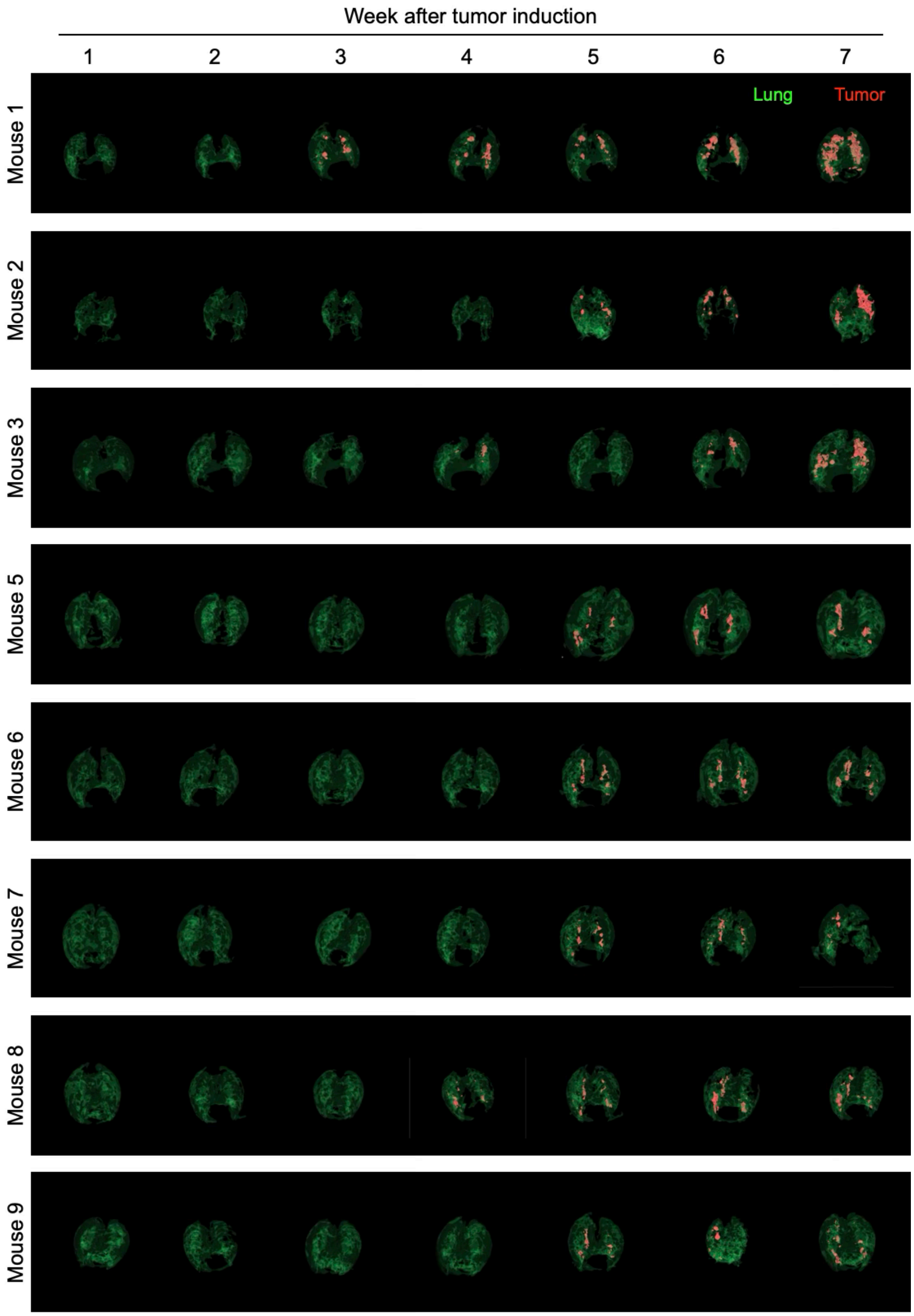

Fig. S15. μCT images of tumor-bearing mice. KP mice underwent μCT imaging weekly after tumor induction. Tumor lesions were delineated (red) from the acquired imaging data. At early LC stages, μCT imaging demonstrated limited sensitivity for tumor burden.

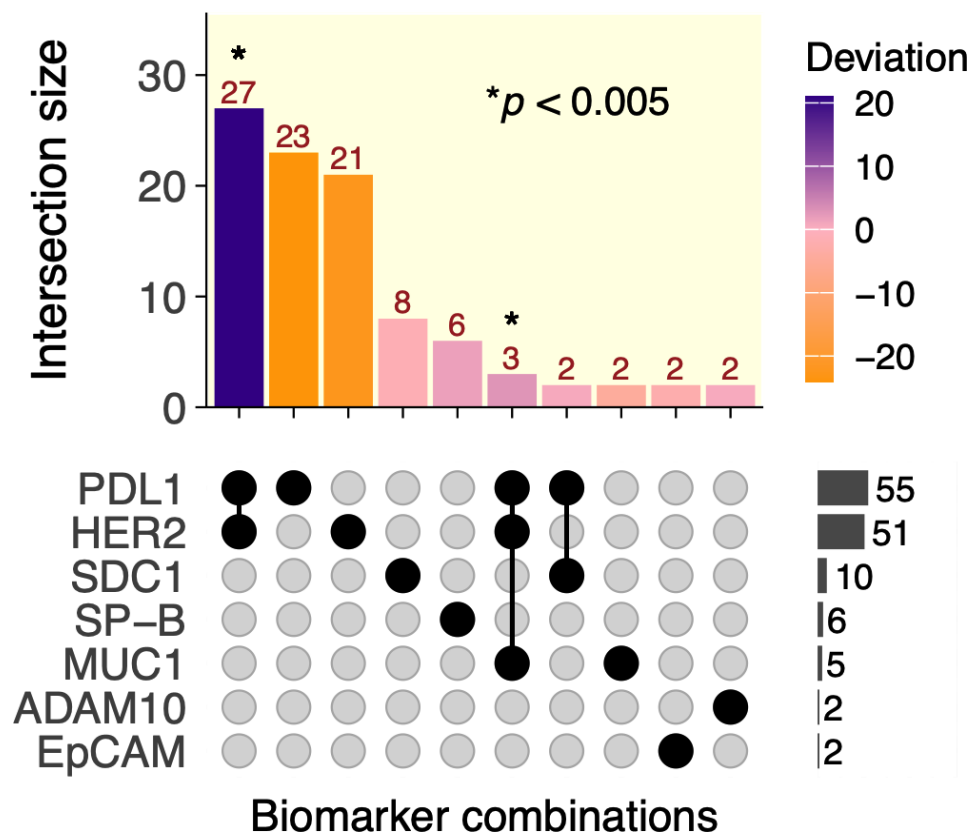

Fig. S16. Upset plot analysis of EVs before tumor induction. Candidate LC markers were expressed at a low level (7%) in total EVs, with minimal co-localization among them.

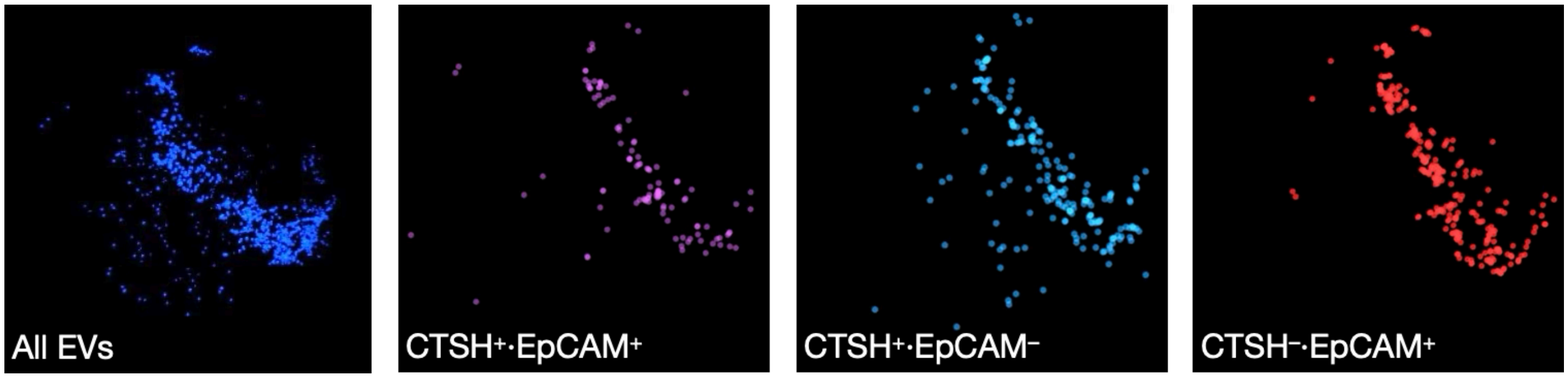

Fig. S17. Distinct EV profiles in week 1 of LC development. CygnusVR visualized EV-profiling data from mice (n = 3) in a 3D t-SNE plot. This visualization revealed a distinct EV population expressing either EpCAM, CTSH, or both.

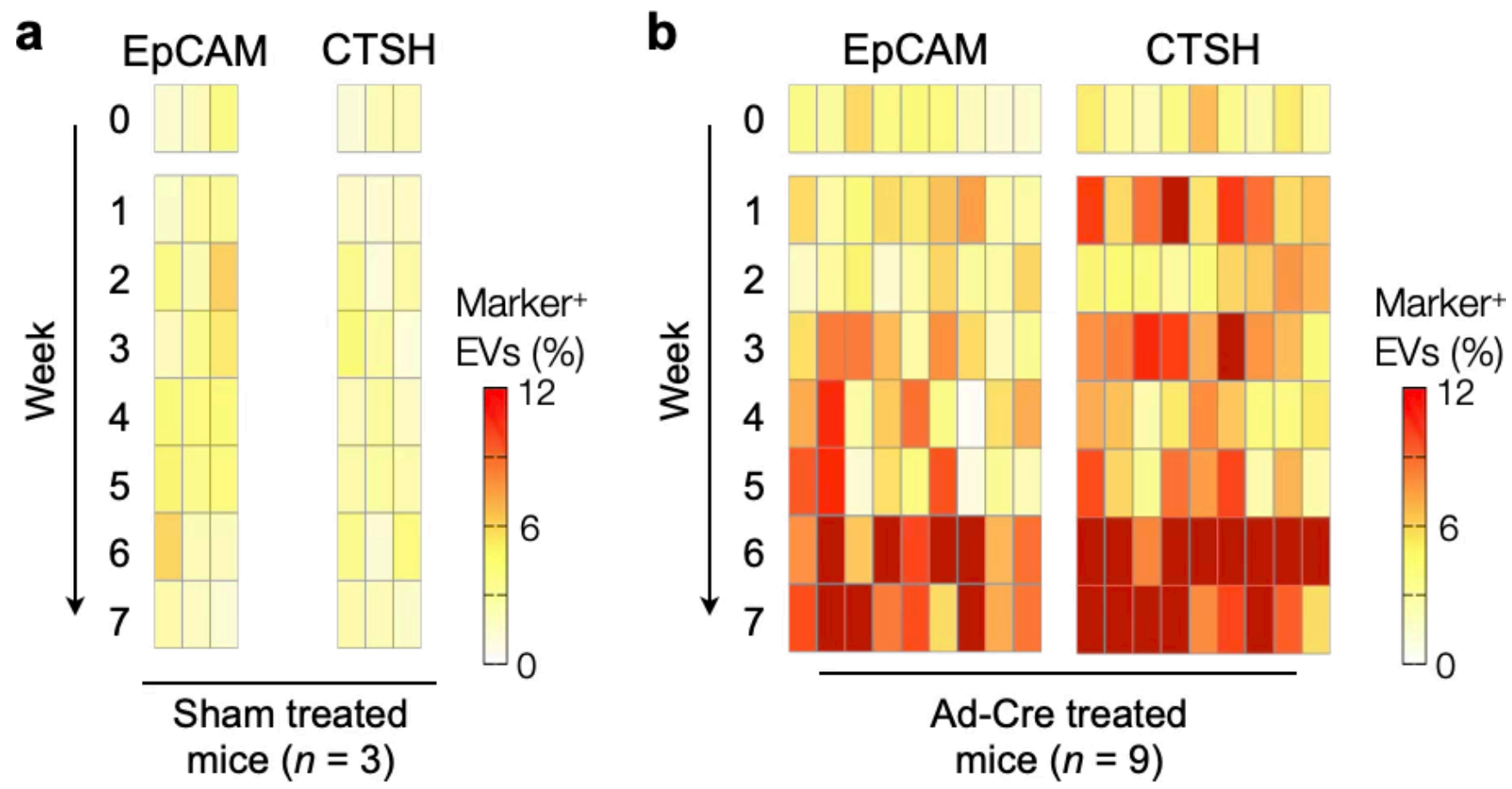

Fig. S18. Longitudinal EpCAM- and CTSH-positive EV fractions in individual mice. Heatmaps show the fractions of EpCAM-positive and CTSH-positive EVs over time in each mouse. **(a)** In sham-treated KP mice receiving intratracheal PBS, EpCAM- and CTSH-positive EV fractions remained low (<4%). **(b)** In Ad-Cre-induced KP mice, EpCAM- and CTSH-positive EV fractions generally increased over time during lung tumor progression.

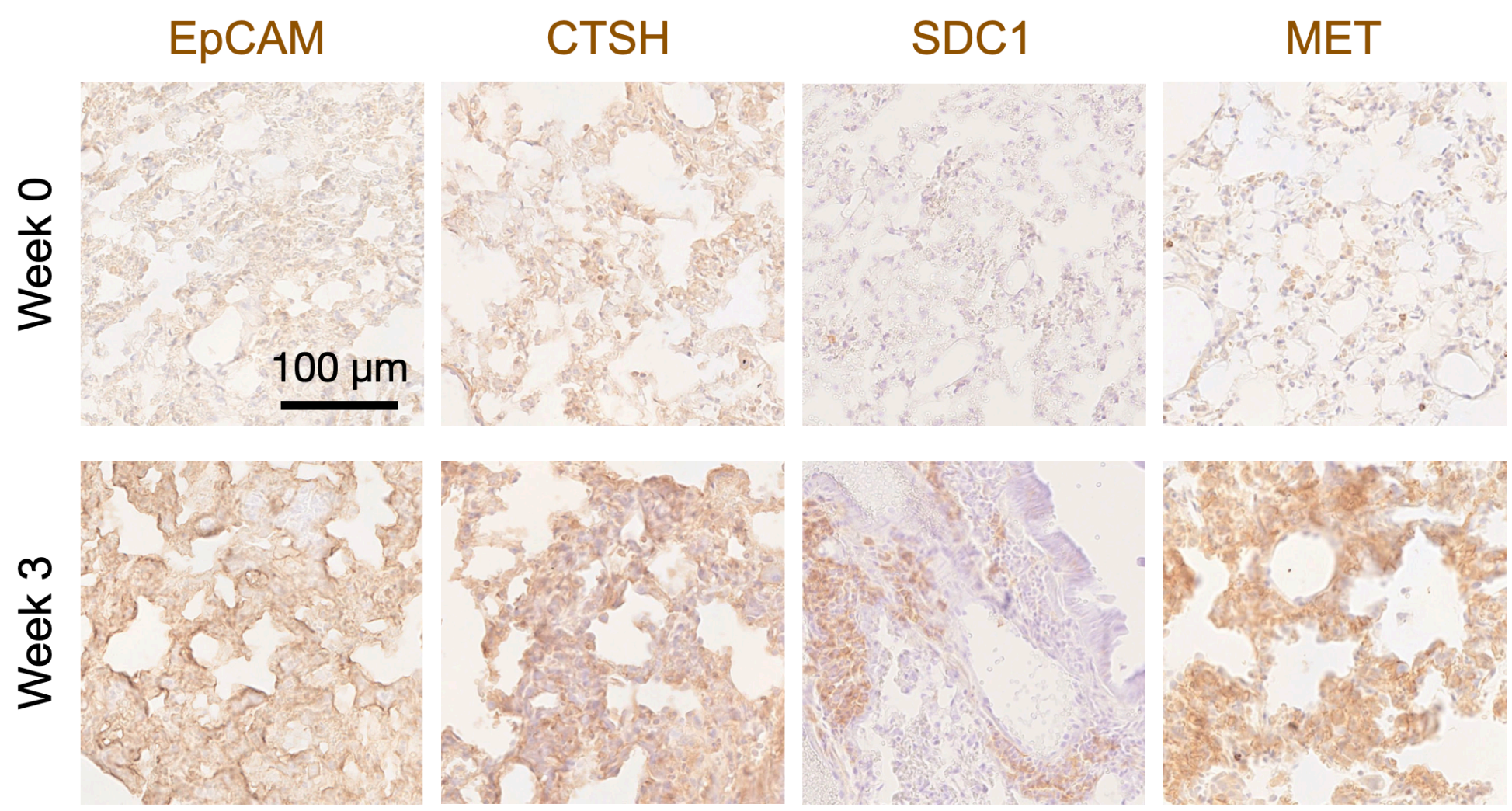

Fig. S19. Immunohistochemical (IHC) staining of tumor tissue. Samples collected at week 3 showed the presence of the cancer markers EpCAM, CTSH, SDC1, and MET.

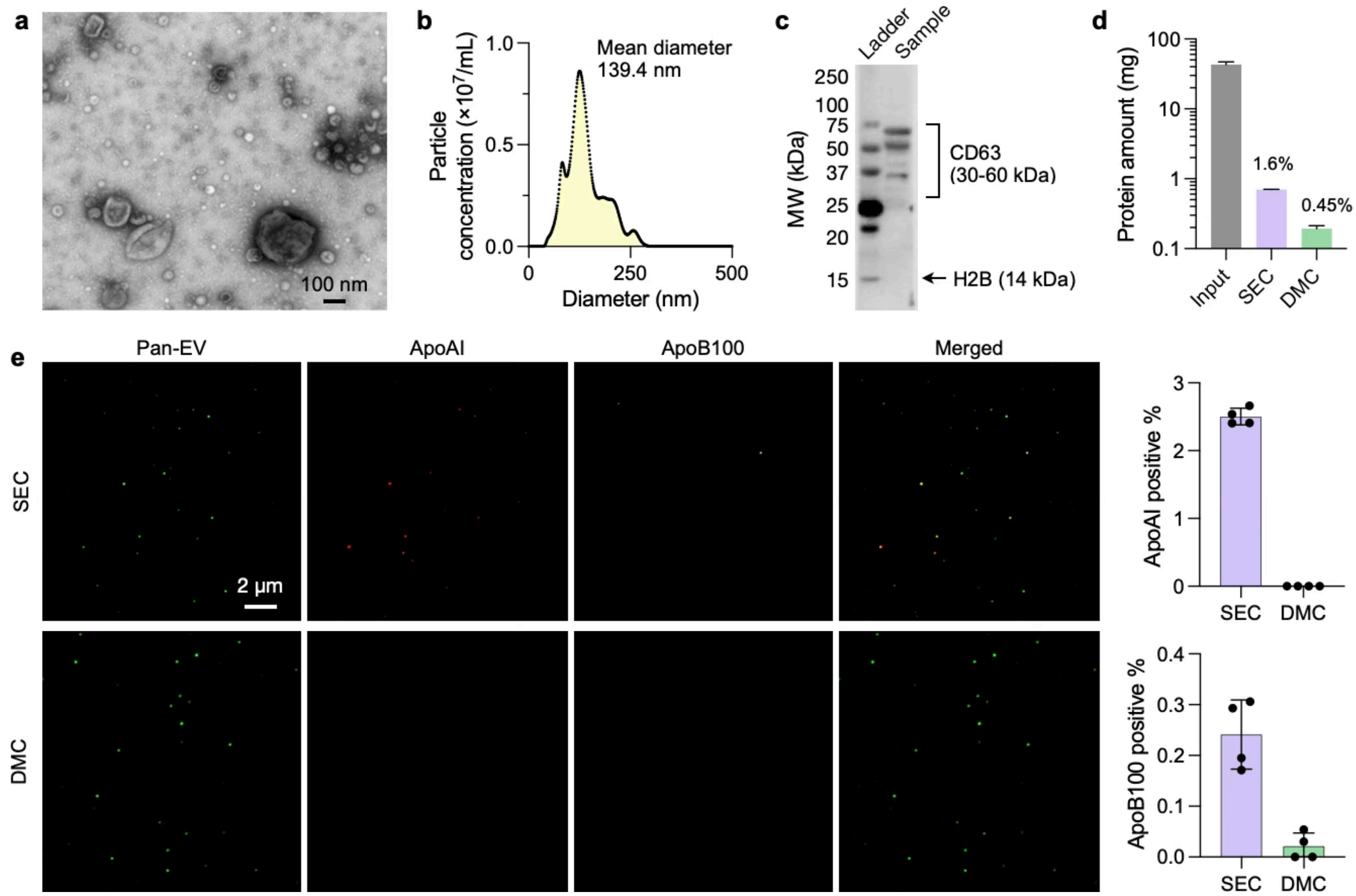

Fig. S20. Characterization of DMC-processed plasma EV preparations. (a) Representative transmission electron microscopy image of EV-like particles isolated from human plasma after dual-mode chromatography (DMC). **(b)** Nanoparticle tracking analysis showing the particle size distribution of DMC-processed plasma. The mean particle diameter was 139.4 nm. **(c)** Western blot analysis. DMC-processed plasma was enriched for the EV-associated tetraspanin CD63 and lacked detectable histone H2B, a nuclear protein used as an EV-negative marker. **(d)** Total protein amount in input plasma and EV preparations isolated by size-exclusion chromatography (SEC) or DMC. Percentages indicate protein remaining relative to input. **(e)** Representative single-EV fluorescence images and quantification of ApoAI- and ApoB100-positive particles in SEC- and DMC-processed plasma samples. Pan-EV signal marks EV-associated particles; ApoAI and ApoB100 were used as markers of HDL and LDL/VLDL-associated lipoprotein particles, respectively. DMC reduced ApoAI and ApoB100 signals compared with SEC, indicating lipoprotein depletion.

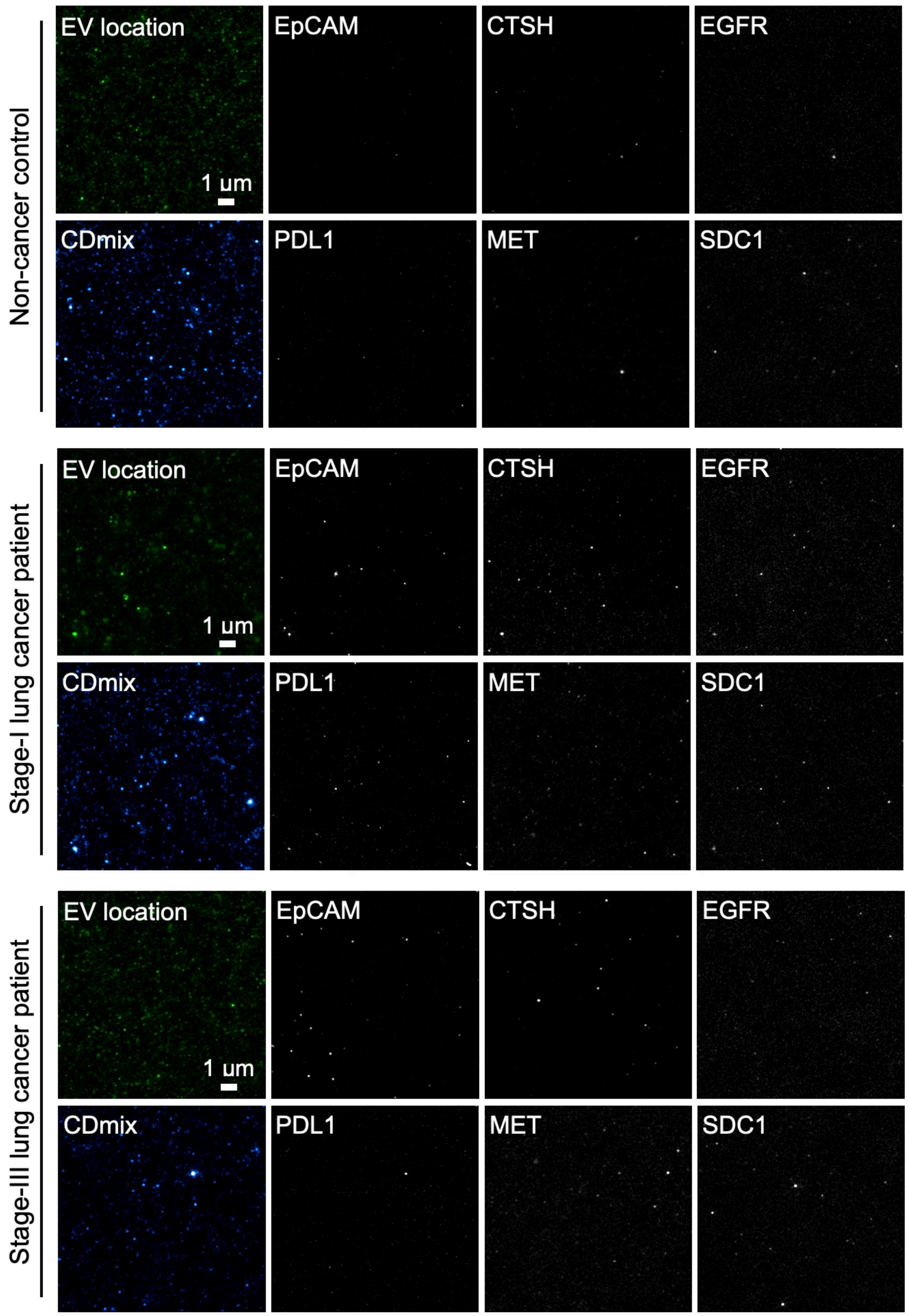

Fig. S21. Profiling of plasma EVs from clinical samples. For universal EV identification (Pan-EV), amine groups on the EV surface were labeled with the NHS-ester form of a fluorescent dye. A mixture of CD9, CD63, and CD81 antibodies (referred to as CD_{mix}) was used to identify tetraspanin-expressing EVs. Six lung cancer (LC) markers were profiled. Representative EV images from non-cancer controls, stage-I LC patients, and stage-III LC patients are shown.

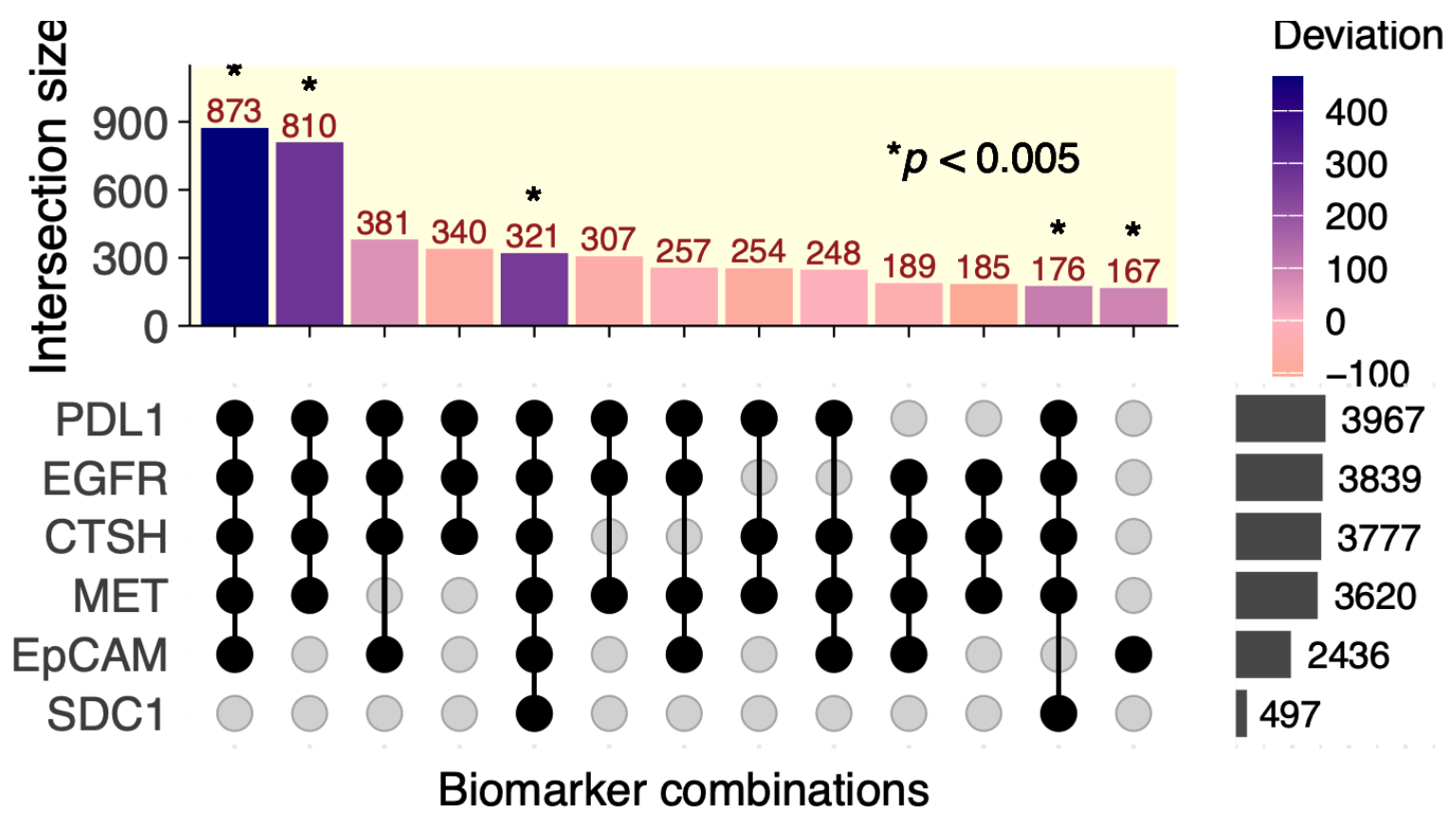

Fig. S22. UpSet and permutation analysis of marker co-expression in a stage IV LC plasma sample. The analysis identified marker combinations with co-expression frequencies exceeding those expected from random marker overlap ($p < 0.005$). Elevated EV counts were observed in multi-marker-positive populations, indicating greater molecular heterogeneity in late-stage LC plasma EVs.

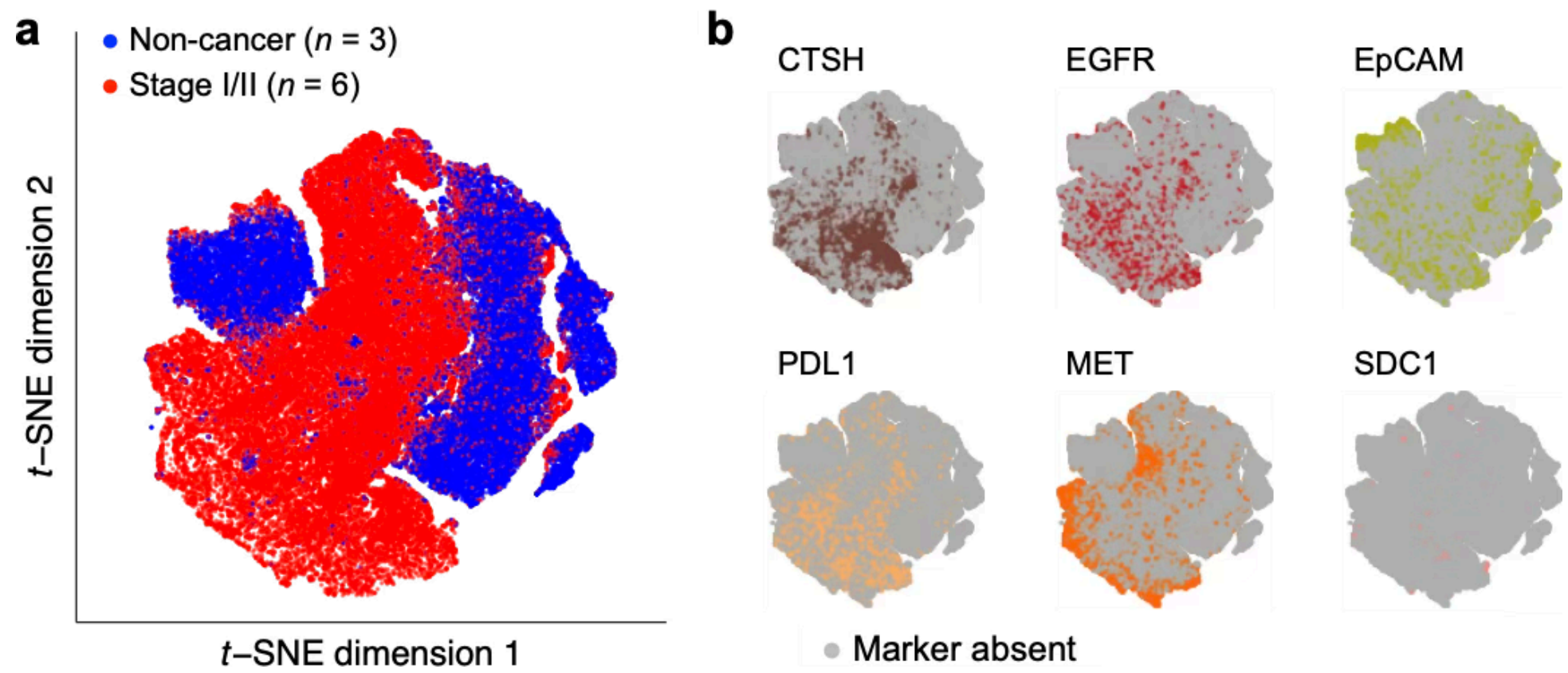

Fig. S23. Single-EV population structure in early-stage lung cancer. (a) *t*-SNE visualization of single-EV profiling data from non-cancer controls ($n = 3$) and patients with early-stage lung cancer (LC; stages I/II, $n = 6$). EV populations were separated according to disease status. **(b)** Marker-expression maps projected onto the *t*-SNE embedding. CTSH, PDL1, and MET were preferentially enriched in EVs from the early-stage LC cohort, whereas EGFR, EpCAM, and SDC1 showed comparatively weaker or less stage-associated enrichment.

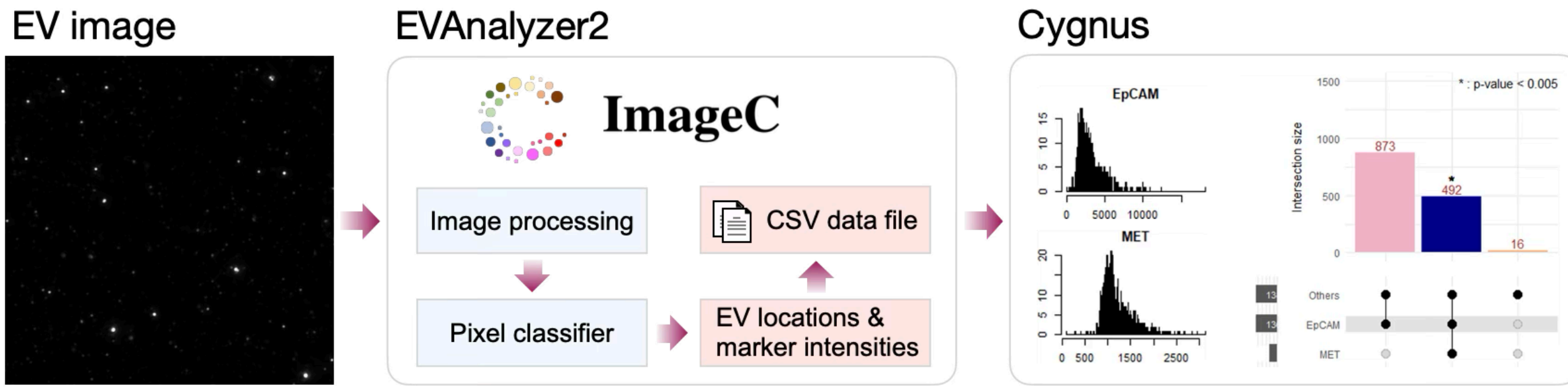

Fig. S24. Cygnus application with other EV-image analysis tools. Cygnus is designed to work seamlessly with existing EV-image analysis software. In this example, EVAnalyzer2 (ImageC) processed a raw single-EV image, identified individual EVs, and generated a tabulated dataset containing marker intensities for each identified EV. Cygnus subsequently imported this data to perform downstream numerical and statistical analyses.

Table S1. Clinical information of cohorts involved in LC detection.

	Non-cancer	LC patients	Total
**Number**	10	20	30
**Age**			
Median	35	61.5	55.5
Range	26–50	46–80	26-80
**Sex**			
Male	5	8	13
Female	5	12	17
**Stage**			
I	-	10	10
II	-	10	10

Table S2. Classification performance of EV marker combinations for distinguishing non-cancer controls from stage I/II lung cancer.

	Marker combination	AUC	Sensitivity	Specificity	Accuracy
1	PD-L1	0.88	0.75	1.00	0.83
	EGFR	0.87	1.00	0.70	0.90
	CTSH	0.84	0.55	1.00	0.70
	EpCAM	0.83	1.00	0.70	0.90
	SDC1	0.79	0.95	0.60	0.83
	MET	0.77	0.95	0.70	0.87
2	PD-L1+CTSH	0.92	0.90	0.80	0.87
	EpCAM+PD-L1	0.91	0.75	1.00	0.83
	PD-L1+EGFR	0.89	0.85	0.90	0.87
	PD-L1+MET	0.89	0.80	1.00	0.87
	MET+EGFR	0.88	1.00	0.70	0.90
	CTSH+EGFR	0.86	0.90	0.70	0.83
	EpCAM+CTSH	0.86	0.65	1.00	0.77
	EpCAM+EGFR	0.85	1.00	0.60	0.87
	CTSH+MET	0.84	0.75	0.80	0.77
	EpCAM+MET	0.83	1.00	0.60	0.87
3	**PD-L1+CTSH+MET†**	0.93	0.85	0.90	0.87
	EpCAM+PD-L1+MET	0.92	0.85	0.90	0.87
	EpCAM+PD-L1+EGFR	0.92	0.75	1.00	0.83
	PD-L1+CTSH+EGFR	0.92	0.90	0.80	0.87
	EpCAM+PD-L1+CTSH	0.91	0.70	1.00	0.80
	CTSH+MET+EGFR	0.90	0.95	0.80	0.90
	EpCAM+CTSH+EGFR	0.89	1.00	0.60	0.87
	PD-L1+MET+EGFR	0.89	0.85	0.90	0.87
	EpCAM+CTSH+MET	0.87	0.75	0.80	0.77
	EpCAM+MET+EGFR	0.87	0.90	0.70	0.83
4	EpCAM+PD-L1+MET+EGFR	0.93	0.80	1.00	0.87
	PD-L1+CTSH+MET+EGFR	0.93	0.85	0.90	0.87
	EpCAM+PD-L1+CTSH+MET	0.93	0.80	1.00	0.87
	EpCAM+PD-L1+CTSH+EGFR	0.91	0.70	1.00	0.80
	EpCAM+CTSH+MET+EGFR	0.87	0.90	0.70	0.83
5	EpCAM+PD-L1+CTSH+MET+EGFR	0.92	0.80	1.00	0.87
6	EpCAM+PD-L1+CTSH+MET+EGFR+SDC1	0.92	0.80	1.00	0.87

† Selected combination with the highest AUC value.

Table S3. Antibodies for mouse plasma-derived EVs.

	Target	Vendor	Origin	Type	Dye
1st cycle	CD63	R&D systems (MAB5417)	Rat	IgG1	Alexa Fluor 647
	CD9	Biolegend (124802)	Rat	IgG2a,κ	Alexa Fluor 647
	CD81	R&D systems (MAB4865)	Rat	IgG1	Alexa Fluor 647
	EGFR	Bioxcell (BE0466)	Mouse	IgG1,κ	Alexa Fluor 488
2nd cycle	MET	Invitrogen (PA5-85951)	Rabbit	polyclonal	Alexa Fluor 647
	SDC1	BD (553712)	Rat	IgG2a,κ	Alexa Fluor 555
	EpCAM	Bioxcell (BE0346)	Rat	IgG2a,κ	Alexa Fluor 488
3rd cycle	ADAM10	R&D systems (FAB946R)	Rat	IgG2a,κ	Alexa Fluor 647
	CTSH	Santa Cruz (sc-398527)	Mouse	IgG1,κ	Alexa Fluor 555
	PDL1	Bioxcell (BE0101)	Rat	IgG2b,κ	Alexa Fluor 488
4th cycle	HER2	R&D systems (FAB6744R)	Rat	IgG1	Alexa Fluor 647
	SP-B	Seven Hills (WRAB-48604)	Rabbit	polyclonal	Alexa Fluor 555
	MUC1	Santa Cruz (sc-53381)	Mouse	IgG1	Alexa Fluor 488

Table S4. Antibodies for human cell line-derived EVs.

	Target	Vendor	Origin	Type	Dye
1st cycle	CD63	Ancell (215-820)	Mouse	IgG1,κ	Alexa Fluor 647
	CD9	BD (555370)	Mouse	IgG1,κ	Alexa Fluor 647
	CD81	Santa Cruz (sc-166029)	Mouse	IgG1,κ	Alexa Fluor 647
	EpCAM	Abcam (ab20160)	Mouse	IgG1,κ	Alexa Fluor 488
2nd cycle	MET	Invitrogen (PA5-85951)	Rabbit	polyclonal	Alexa Fluor 647
	SDC1	BD (550804)	Mouse	IgG1,κ	Alexa Fluor 555
	EGFR	Bioxcell (BE0278)	Mouse	IgG1,κ	Alexa Fluor 488
3rd cycle	ADAM10	Abcam (ab1997)	Rabbit	polyclonal	Alexa Fluor 647
	CTSH	Santa Cruz (sc-398527)	Mouse	IgG1,κ	Alexa Fluor 555
	PDL1	Bioxcell (BE0285)	Mouse	IgG1,κ	Alexa Fluor 488
4th cycle	SP-B	Seven Hills (WRAB-48604)	Rabbit	polyclonal	Alexa Fluor 647
	MUC1	Fitzgerald (10-M93A)	Mouse	IgG1,κ	Alexa Fluor 555
	HER2	Genetech (Herceptin)	Human	IgG	Alexa Fluor 488

Supporting Movie Legends

Movie S1. Multi-Image Alignment Code Demonstration
This movie demonstrates how the image processing plugin operates. There are three main steps. i) *CBD_Drawline_images* defines fiduciary points for image alignment, ii) *CBD_stack_image* aligns and stacks the images into a unified dataset, and iii) *CBD_ComDet_Auto* performs automated particle detection and colocalization. It outputs results as CSV files and saves annotated images together with the analysis results. This plugin can be an alternative to AIVIA-based segmentation.

Movie S2. CygnusVR Immersive View and Clustering Scene (Human Dataset)
This movie shows the CygnusVR immersive 3D environment, featuring the human dataset in both immersive view and clustering scene, with actual user interaction. i) In the immersive view, users can analyze and compare multiple results within a spherical room. ii) In the clustering scene, users can manipulate 3D clustering results, including marker or group selection, as well as zooming and scaling.

Supplementary Note — Multi-image Alignment Code

We have developed a suite of ImageJ plugins to facilitate the analysis of cyclic EV images. These plug-ins perform background subtraction, multi-image alignment, image stacking, and particle detection (see **Movie 1**). The processes allow researchers to transform their raw data into a clean, unified dataset ready for further analysis (e.g., Cygnus). The plugins are available for download at https://github.com/kylie0914/Multi-Image-Alignment-Code.

A. Overall workflow

Users should specify the directory containing the raw input images (TIF format) and follow the on-screen prompts during the interactive steps. The plugins automatically generate dedicated output directories for processed results, maintaining an organized data architecture throughout the analysis pipeline. The following sequential steps are performed:

CBD_iter_SubtBG.ijm	Perform background subtraction on raw fluorescence images.
CBD_Drawline_images.ijm	Draw alignment lines on the first image and apply them to others.
CBD_Stack_images.ijm	Align and stack the images to create a unified dataset.
CBD_ComDet_Auto.ijm	Detect particles and analyze colocalization in the stacked images.

B. Background subtraction (CBD_iter_SubtBG.ijm)

This plugin enhances signal contrast by removing background signals from fluorescence images using a rolling-ball algorithm.

Function	It iteratively processes all .tif images in the input folder, applies background subtraction with a 50-pixel rolling radius, and saves the cleaned images in a BackgroundSubtracted directory
Usage	Users specify a folder containing raw .tif images, and the plugin outputs background-subtracted images in a designated subfolder.

C. Image alignment (CBD_Drawline_images.ijm)

This plugin allows users to define alignment markers (**Fig. N1**) by drawing reference lines on fluorescence images.

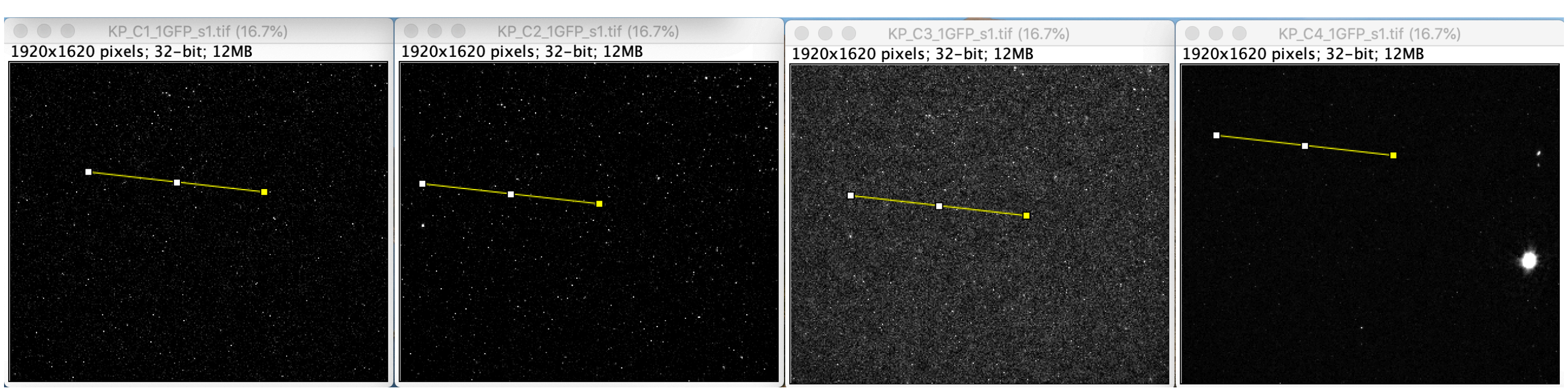

Fig. N1. Defining fiduciaries for image alignment. For image alignment, users identify fiducial markers within a selected image from each cyclic acquisition.

Function	The plugin sequentially loads images from the input folder. The user draws a straight line connecting two fluorescence markers on the first image, and the plugin replicates this line on subsequent images. The updated images are saved in an output directory.
Usage	Users input a folder with raw .tif images, and the plugin outputs images with alignment lines. Interaction is required to draw the initial alignment line.

D. Image stacking (CBD_Stack_images.ijm)

This plugin aligns and stacks fluorescence images (**Fig. N2**) to create a unified dataset for analysis.

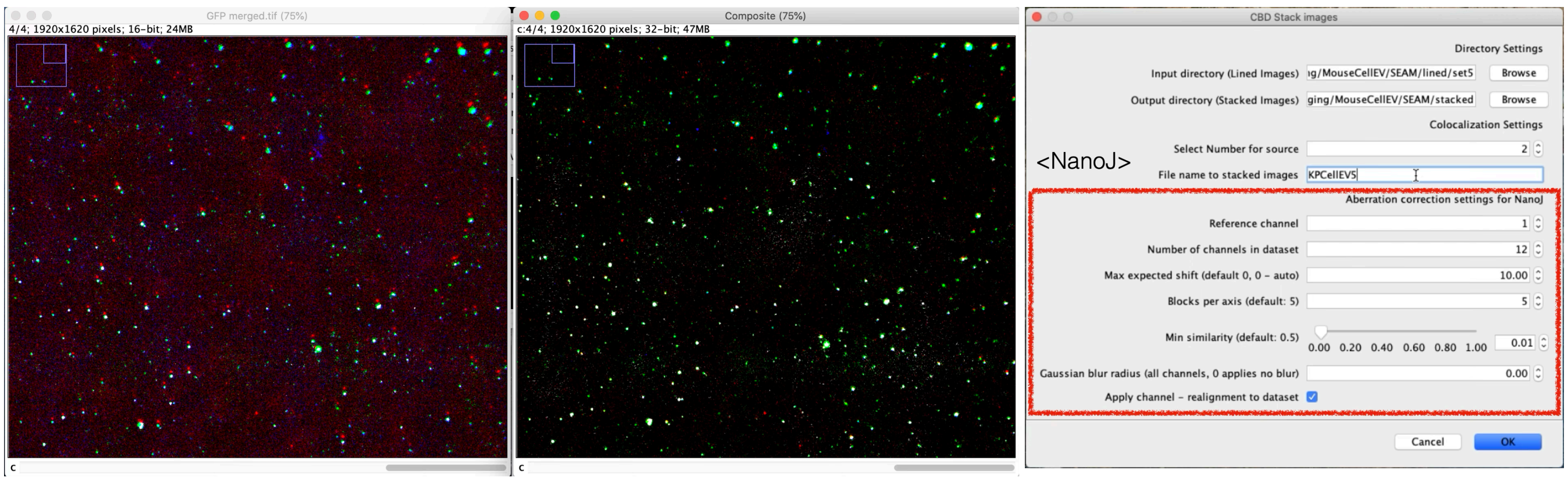

Fig. N2. Image stacking examples. The figure illustrates image stacking, showing images prior to alignment (Left), following alignment and stacking (Middle), and the corresponding plugin interface (Right). The plugin additionally performs aberration corrections during the image alignment process.

Function	It aligns each image to a reference image using predefined alignment lines, stacks the aligned images into a hyperstack, and corrects for channel misalignment if necessary. The final stacked image is saved as a .tif file.
Usage	Input is a folder containing images with alignment lines, and output is a single .tif file containing the stacked images. Adjustable parameters include the reference channel (default: 1) and the number of channels (default: 12).

E. Particle detection and colocalization (CBD_ComDet_Auto.ijm)

This plugin automates particle detection and colocalization analysis in stacked images.

Function	It detects particles based on user-defined parameters (e.g., particle size, intensity threshold, and maximum distance for colocalization), outputs results as CSV files, and saves annotated images and analysis results.
Usage	Input is a stacked .tif image, and output includes detection results in CSV format and processed images. Parameters include particle size (default: 3.00 pixels), intensity threshold (default: 5.00 SD), and maximum colocalization distance (default: 4.00 pixels).

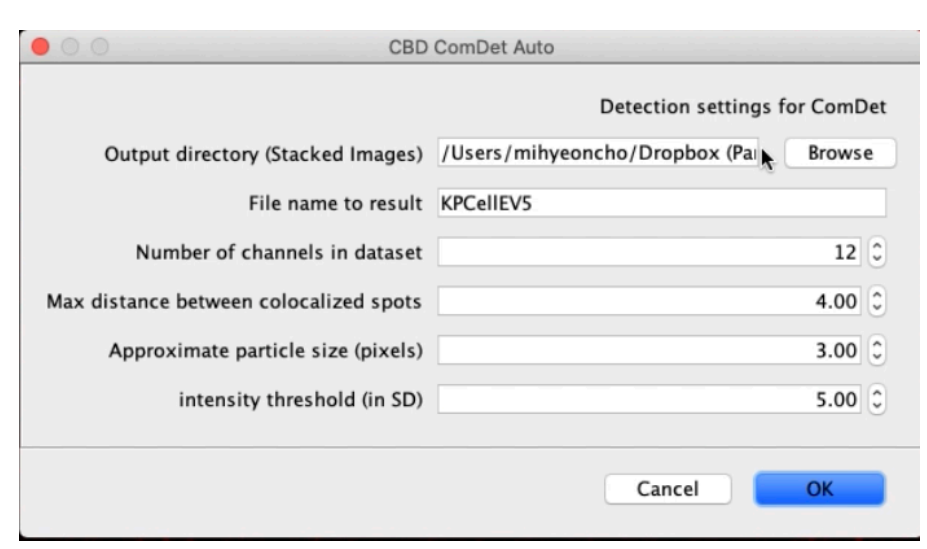

Fig. N3. Interface for EV detection. The plugin uses ComDet to identify EVs within a given image stack.
